# USP39 stabilizes β-catenin by deubiquitination and suppressing E3 ligase TRIM26 pre-mRNA maturation to promote HCC progression

**DOI:** 10.1038/s41419-023-05593-7

**Published:** 2023-01-27

**Authors:** Weiwei Wang, Yongbin Lei, Gongye Zhang, Xiaomei Li, Jiahui Yuan, Tingting Li, Wei Zhong, Yuqi Zhang, Xuemei Tan, Gang Song

**Affiliations:** Cancer Research Center, School of Medicine, Xiamen University, Xiamen, China

**Keywords:** Cell signalling, Oncogenes, Molecular biology

## Abstract

Ubiquitin-specific protease 39(USP39) plays an important role in modulating pre-mRNA splicing and ubiquitin-proteasome dependent proteolysis as a member of conserved deubiquitylation family. Accumulating evidences prove that USP39 participates in the development of hepatocellular carcinoma (HCC). However, little is known about the mechanism especially deubiquitinating target of USP39 in regulating hepatocellular carcinoma (HCC) growth. Here, we prove that USP39 promotes HCC cell proliferation and migration by directly deubiquitin β-catenin, a key molecular of Wnt/β-catenin signaling pathway whose abnormal expression or activation results in several tumors, following its co-localization with USP39. In this process, the expression of E3 ligase TRIM26, which is proved to restrain HCC in our previous research, shows a decreasing trend. We further demonstrate that TRIM26 pre-mRNA splicing and maturation is inhibited by USP39, accompanied by its reduction of ubiquitinating β-catenin, facilitating HCC progression indirectly. In summary, our data reveal a novel mechanism in the progress of HCC that USP39 promotes the proliferation and migration of HCC through increasing β-catenin level via both direct deubiquitination and reducing TRIM26 pre-mRNA maturation and splicing, which may provide a new idea and target for clinical treatment of HCC.

## Introduction

As the fifth most common cancer in the world, hepatocellular carcinoma (HCC) endangers human health seriously and causes the second highest mortality rate in the world [1–4]. In Asian countries, especially China, the death rate from HCC is much higher than in western countries [5]. Due to its insidious onset, strong invasiveness, insensitivity to chemotherapy, poor prognosis and other characteristics, HCC brings great challenge in clinical treatment. Therefore, the discovery of new molecular targets for HCC treatment becomes particularly important, which can provide a broader prospect for the treatment.

Emerging studies have shown that Wnt/β-catenin signaling pathway plays an important role in EMT and tumor progression. β-catenin activation is observed in 20–35% of hepatocellular carcinoma (HCC) cases, which also indicates that β-catenin accumulation is closely associated with HCC progression and poor prognosis [6–8]. Although epigenetic alterations in DNA methylation [9], histone modifications [10], or noncoding RNAs have been proved to regulate abnormal Wnt/β-catenin activation in HCC [11]. However, little is known about the regulation to β-catenin through ubiquitinating and deubiquitinating ways during HCC progression.

The ubiquitin-specific peptidase 39 (USP39) belongs to the family of deubiquitinating enzymes (DUB), containing the amino-terminal zinc-finger ubiquitin-binding domain (ZNF-UBP) and the carboxy-terminal ubiquitin hydrolase domain (USP) [12]. USP39 is originally found as a regulator of RNA splicing in the tumor progression. Studies confirm the importance of USP39 on splicing regulation and its requirement for the survival of KRAS-dependent tumors [13]. USP39 can also promote the development of glioma by inducing the maturation of TAZ mRNA to increase its protein level [14].

It was once thought that USP39 has no DUB activity due to the lack of conserved active sites (cysteine, histidine, and aspartic acid) in the deubiquitinating domain [15]. However, subsequent studies have proved that USP39 could regulate the stability of DNA damage-related protein CHK2 [16], stabilize SP1 and prolong its half-life period [17], or stabilize STAT1 in the type I IFN signaling pathway thereby promoting innate antiviral immunity through its deubiquitinating function [18]. In addition, our previous studies also showed that USP39 promotes HCC progression by the deubiquitination of ZEB1 [19]. No denying many studies have proved that USP39 plays a cancer-promoting role in HCC [20–22], however, the regulation of splicing and deubiquitination that USP39 played in the development of HCC remains to be further studied.

TRIM26 belongs to the TRIM family of E3 ligases, participating in diverse cellular processes such as cell proliferation, DNA repair, and innate immunity [23–26]. As a tumor suppressor, TRIM26 shows a decreasing tendency in a variety of cancers such as papillary cancer [27], non-small cell lung cancer [28], nasopharyngeal carcinoma [29] and HCC [30]. Our previous study also showed that TRIM26 suppresses HCC by ubiquitinating ZEB1, a crucial driver of tumor by inducing the EMT in tumor epithelial cells. In consideration of ZEB1 deubiquitination by USP39, we wondered if there were other protein participates in HCC development, which could be simultaneously regulated by USP39 and TRIM26. Interestingly, we find a close relationship between USP39, TRIM26 and the crucial member of Wnt signaling pathway that is β-catenin.

In this work, we investigate the functional role of USP39 in the development of HCC in vitro, and reveal that USP39 has dual functions in regulating β-catenin in HCCs. We first verify USP39 directly interact with β-catenin protein and further find that USP39 can stabilize β-catenin protein by deubiquitylation. In detail, on one hand, USP39 decreases β-catenin degradation through its deubiquitination function to promote HCC progression. On the other, USP39 promotes the proliferation and invasion of HCC cells by inhibiting splicing of TRIM26, thus affecting the ubiquitin of β-catenin induced by TRIM26. Collectively, our data suggest that USP39 has deubiquitinating and RNA splicing activity, promoting HCC progression by directly or indirectly regulating β-catenin. These findings provide a novel mechanism for understanding the progression of HCC, and may provide inspiration on further finding promising therapeutic targets against human HCC.

## Materials and methods

### Cell lines and cell culture

Human hepatoma carcinoma cell lines SK-hep-1, PLC/PRF/5, and HepG2 were obtained from the Shanghai Cell Bank of the Chinese Academy of Sciences, China. SK-hep-1 was cultured in MEM (Hyclone, Logan, Utah, USA) supplemented with 10% fetal bovine serum (FBS; Gbico, Massachusetts, USA). PLC/PRF/5 and HepG2 were cultured in DMEM (Hyclone) supplemented with 10% fetal bovine serum (FBS; Gbico). All cells were maintained at 37 °C in a humidified chamber with 5% CO_2_. All cell lines were authenticated (National Collection of Authenticated Cell Cultures, Shanghai, China) and were verified to be mycoplasma negative.

### Plasmid construction and gene transduction

Plasmids to knock down USP39 or TRIM26 were purchased from Genechem (Shanghai, China). The sequences of the shRNA for human TRIM26 mRNA are as follows: shRNA#1 GATGGATATGACGACTGGGAA and shRNA#2 GCTGCTGAGAGACTTGGAATA. Target sequences for human USP39 mRNA are as followed: shRNA#1 TTCCAGACAACTATGAGAT and shRNA#2 TTTGGAAGAGGCGAGATAA. The β-catenin knockdown plasmid was constructed on the plko.1 plasmid. Target sequences for human β-catenin mRNA as followed: shRNA#1 GGTTAATAAGGCTGCAGTTAT and shRNA#2 GCTTATGGCAACCAAGAAAGC. Plasmids were transferred into HEK 293T cells by PEI to generate lentivirus. After 48 h, the supernatant was collected and added onto cells in culture supplemented with 8 μg/ml polybrene.

For ectopic expression of TRIM26 and USP39, Containing the full-length trim26 fragment of pcmv-N-Myc plasmid (Myc-TRIM26) was constructed and sequenced. Then these plasmids were transferred into HCC cells via liposomes (Yeasen, Shanghai, China).

### Subcellular fractionation

Nuclear and cytoplasmic fractions from different cell populations were isolated using Nuclear and Cytoplasmic Extraction Reagents according to the manufacturer’s instructions (Beyotime, Shanghai, China). Nuclear and cytoplasmic proteins were analyzed by western blotting. Tubulin and lamin B were used for normalization.

### Western blot analysis

Cells were lysed with RIPA lysis and Extraction buffer containing a protease inhibitor cocktail (ROCHE, Basel, Switzerland), Protein lysates(20 μg) were separated by SDS-polyacrylamide gel electrophoresis and transferred onto polyvinylidene fluoride membranes (EMD Millipore Billerica, USA). Membrane was blocked in 5% skim milk and incubated overnight with specific primary antibodies at 4 °C followed by incubation with secondary antibodies for 1 h at room temperature. Proteins on membranes were visualized using Electrochemiluminescence (Thermo Fisher Scientific, USA) and signals were detected with the ChemiDocTM MP Imaging System (Bio-Rad Laboratories, California, USA). The primary antibodies used in Western blotting are as follows: anti-USP39 (ab131244, 1:1000) (Abcam, Cambridge, England), TRIM26 (27013-1-AP, 1:1000) (proteintech, Chicago, USA), non-phosphor (active) b-catenin (#8814, 1:1000) (CST, Boston, USA) and β-actin (1:40,000) (Sigma-Aldrich, Missouri, USA).c-myc (1:1000) (proteintech), cyclin D1(1:1000) (proteintech).

### Immunofluorescence

Cells were cultured in six-well plates, and fixed with 4% paraformaldehyde for 10 min at room temperature. Gently wash the sample three times with PBS, then permeabilized with 0.2% Triton X-100 (Sigma-Aldrich, USA) for 10 min on ice. Samples were blocked with 5% bovine serum albumin for 1 h, and incubated with primary antibody at 4 °C overnight. Primary antibody was detected with fluorescence-conjugated rabbit/mouse secondary antibody (Invitrogen, California, USA) for 1 h in dark conditions. Cell nuclei were stained with DAPI (Invitrogen, USA) for 10 min. Images were obtained under fluorescence microscope (Olympus, Tokyo, Japan).

### Reverse transcription PCR

Total RNA was isolated from cells using TRIzol reagent. (Takara, Tokyo, Japan). Isolated total RNA was quantified and transcribed into cDNA using a reverse transcription kit (yesen, Shanghai, China). Quantitative RT-PCR was used to measure mRNA expression levels of the tested genes. GAPH was used as a control, and the relative fold change of the target gene was calculated by the 2−ΔΔCq method. Primer sequence information for specific genes is listed in Supplementary Materials (Table 1).

### Cell viability assay

HCC cell viability was detected using a cell proliferation assay kit (Beyotime, China). Cells were seeded into 96-well plates with or without ICG-001at a density of 5000 cells/well. At indicated time points, 20 μL of MTT reagent (Promega, Madison, Wis, USA) was added to each well and then incubated for 4 h at 37 °C. The cell proliferation curves were determined by measuring optical density (OD) at a wavelength of 570 nm.

### Transwell assay

HCC cells were resuspended in serum-free medium and seeded at a density of 50,000 per well in the upper chamber of a 24-well plate. Medium containing 20% serum was added to the lower chamber and ICG-001 reagents were added to both of upper and lower chamber. After the cells were incubated for 24 h, the remaining cells on the upper surface of the chamber were removed by cotton swabs. Cells that migrated to the lower surface of the chamber were fixed with 4% paraformaldehyde and stained with crystal violet. The chamber was observed and photographed under a microscope, and each experiment was repeated three times.

### Colony formation assay

Cells were seeded in 6-well plates at a density of 500 cells/well with ICG-001(yeasen). Cells were cultured in cell chambers for 2 weeks then cells were fixed with 4% paraformaldehyde and stained with crystal violet (Sigma-Aldrich, USA). Colony were counted and normalized to the control. Each experiment was repeated three times.

### Wound-healing assay

Cells were seeded in 6-well plates and scratch was made by a 20 μl pipette tip after cells were grown to 80% confluence. Cells were then incubated in medium containing 2% FBS and ICG-100. Gap size was measured 24 h later. Cell migration was normalized to the control and each experiment was repeated three times.

### RNA-binding protein immunoprecipitation assay

Cells were lysed by RIP lysate containing RNase (Roche) out and Protease inhibitor cocktail (Roche), and 5% cell lysate was used as input. The remaining cell lysate was incubated with protein A/protein G agarose beads overnight at 4 °C. The agarose beads were washed three times, and total RNA was extracted according to the method for cell total RNA extraction. Total RNA was reversed into cDNA, and mRNA levels were quantified by qRT-PCR.

### Immunoprecipitation assay

Immunoprecipitation experiments were performed according to previously established methods [31]. Cells were lysed in RIPA buffer (yeasen) containing PMSF (yeasen), and 5% cell lysate was used as input. The remaining cell lysate was incubated with protein A/protein G agarose beads for 4 h at 4 °C. After that, the agarose beads were washed three times, added with 2× SDS-loading buffer and boiled for 5 min. Samples were analyzed by western blot.

### GST pull-down assay

The GST-tagged plasmid was transformed into *E. coli* BL21 and purified. Cell lysate was obtained by expressing HA-β-catenin in HEK 293T cells, and the cell lysate and purified GST-tagged protein were incubated at 4 °C for 4 h. Add SDS-loading buffer to the sample and boil for 5 min, then detect protein ubiquitination by western blotting.

### In vivo ubiquitination assay

Cells were transfected with Flag/HA-Ubiquitin and indicated plasmids for in vivo ubiquitination assay, which was performed as previously described [32]. In short, cells with indicated treatment were incubated with MG132 for 6 h and lysed with denaturing lysis buffer. The cell lysates were boiled for 10 min, renatured with bovine serum albumin washing buffer and subjected to IP with the appropriate antibody. The immunoprecipitates were analyzed by western blot.

### Immunohistochemistry

Paraffin sections (4 μm thick) were first incubated in an oven at 60 °C for 2 h, then deparaffinized with xylene and rehydrated through gradient ethanol immersion. Boiled for antigen retrieval with 0.01 mol/L citrate buffer solution (pH 6.0) for 30 min. Endogenous peroxidase blocking reagent (Cell Marque, Rocklin, CA) was added for 10 min to block non-specific antibody binding. Sections were also blocked in 1% fetal calf serum for 10 min, and then incubated with the primary antibody in humidified chamber overnight at 4 °C, and treated with a streptavidin-peroxidase-conjugated second antibody (Fuzhou Maixin Biotech. Co., Ltd, China) for 30 min at room temperature. The immune complexes were visualized with a Vectastatin DAB kit (Vector Laboratories). Staining intensity scoring was performed in a blinded manner by two uninvolved scientists.

### In vivo experiments

The nude mouse xenograft model: 6–8-week-old male nude mice (18–20 g) were purchased from the National Model Animal Center of Nanjing University and maintained in a specific pathogen-free environment with free access to food and water and a 12-h light/dark cycle. The mice were randomly divided into a control group (*n* = 6), shTRIM26 group (*n* = 6) and shTRIM26+shb-catenin group (*n* = 6). SK-hep-1 cells (1 × 10^6^) stably transduced with shTRIM26 and shTRIM26+shβ-catenin were injected subcutaneously into the flanks of 6–8-week-old male BALB/c nude mice, respectively. Tumor length (*a*) and width (*b*) were measured with a vernier caliper every 3–4 days, and then tumor volume was calculated using the formula: 1/2*ab*^2^. The mice were sacrificed on the 30th day after injection, and tumors were isolated. Tumor tissues were stored at −80 °C and fixed with 4% paraformaldehyde for immunohistochemistry (IHC).

For extrahepatic metastasis model: 6–8-week-old male nude mice (18–20 g) were purchased from the National Model Animal Center of Nanjing University and maintained in a specific pathogen-free environment with free access to food and water and a 12-h light/dark cycle. The mice were randomly divided into a control group (*n* = 4), shTRIM26 group (*n* = 4), control+ICG-001 group (*n* = 4), shTRIM26+ICG-001 group (*n* = 4). 1 × 10^6^ Sk-hep-1 cells were injected into nude mice of each group through the caudal vein, and the mice were sacrificed 40 days later to observe liver metastasis. No blinding was done for animal studies.

### Statistical analysis

Each experiment was repeated independently at least three times. Continuous variables were presented as mean and standard deviation (SD). Unpaired two-tailed student’s *t*-test or one-way analysis of variance was employed for comparison of continuous variables. All Analyses were per- formed using GraphPad Prism 8 software (GraphPad Software, USA). A *P* value of <0.05 was considered statistically significant. Asterisks (*, **, *** and****) stand for *P* < 0.05, *P* < 0.01, *P* < 0.001 and *P* < 0.0001, respectively.

## Result

### USP39 increases proliferation and migration of HCC cells through the Wnt/β-catenin pathway

**Fig. 1:**
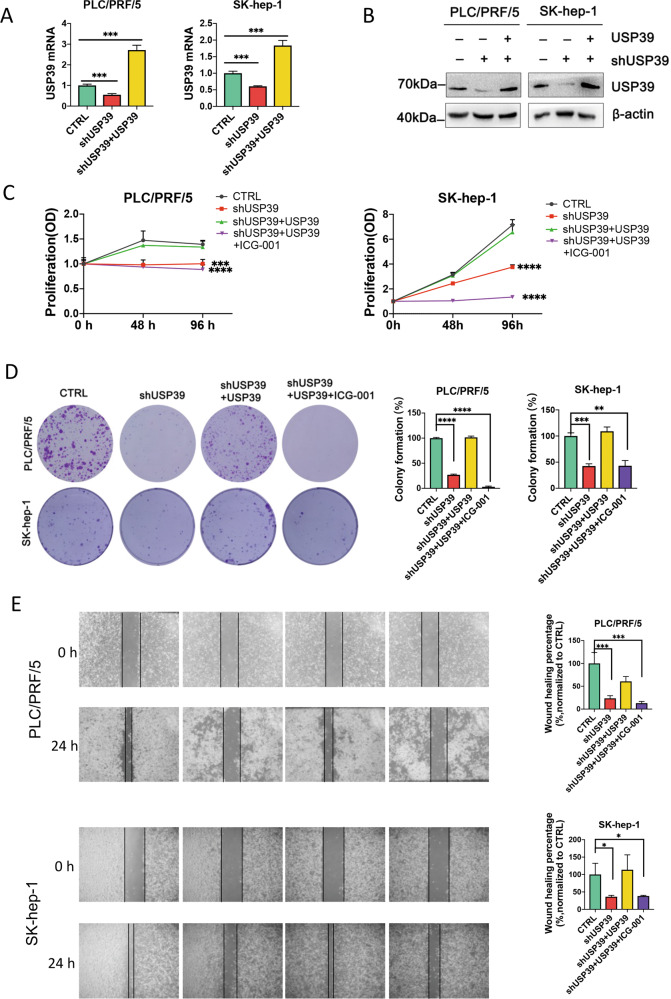
USP39 increases proliferation and migration of HCC through the wnt signaling pathway. **A** The expression of USP39 mRNA was analyzed by RT-PCR in PLC/PRF/5 and SK-hep-1 cells. **B** Western blot confirmed the expression of USP39 after cells were knocked down and overexpressed **C** The effects of USP39 on the proliferation of HCC cells (PLC/PRF/5 and SK-hep-1 cells) were examined by MTT assay in the presence or absence of ICG-001. **D** Colony formation assays were performed in HCC PLC/PRF/5 and SK-hep-1 cells (The colony numbers were normalized to controls and expressed as a percentage). **E** Wound-healing assays were performed to determine the effect of USP39 on HCC cells (PLC/PRF/5 and SK-hep-1 cells) migration in the presence or absence of ICG-001. All the data are representative of at least three independent experiments and presented as the means ± SD. (*p < 0.05; **p < 0.01; ***p < 0.001; ****p < 0.0001 v.s. control by one-way ANOVA /two-way ANOVA or Student’s *t*-test).

To elucidate whether USP39 regulate β-catenin expression, cell proliferation, cell cycle, and tumor growth in HCC cells, knockdown and overexpression of USP39, β-catenin inhibitor ICG-001 were performed in HCC cell lines (SK-hep-1 and PLC/PRF/5). The efficiency of knockdown and complementation of USP39 was confirmed by quantitative real‐time PCR (qRT‐PCR) (Fig. 1A) and western blotting (WB) (Fig. 1B) respectively. HCC cell proliferation ability was assessed by MTT and colony formation assays. MTT results indicated that knockdown of USP39 reduced the proliferation of HCC cells compared with the control group, while replenishment of USP39 restored the proliferation of HCC cells. Nevertheless, USP39 replenishment failed to restore HCC cell proliferation when the wnt/β-catenin pathway was blocked by ICG-001 (Fig. 1C). Colony formation assays demonstrated that USP39 knockdown remarkably restrained colony formation ability of HCC cells, and this effect was recovered by replenishment of USP39. However, colony formation ability was inhibited again when treated with ICG-001 (Fig. 1D). In addition, wound-healing assays showed that silencing USP39 significantly decreased the migration of HCC cells, replenishing USP39 rescued the migration ability impaired by silencing USP39, and the Wnt pathway inhibitor ICG-001 reverted this phenomenon. (Fig. 1E). These data demonstrated that USP39 increases the proliferation and migration of HCC cells through the Wnt/β-catenin pathway.

### Loss of USP39 leads to a decrease of β-catenin in HCC

**Fig. 2:**
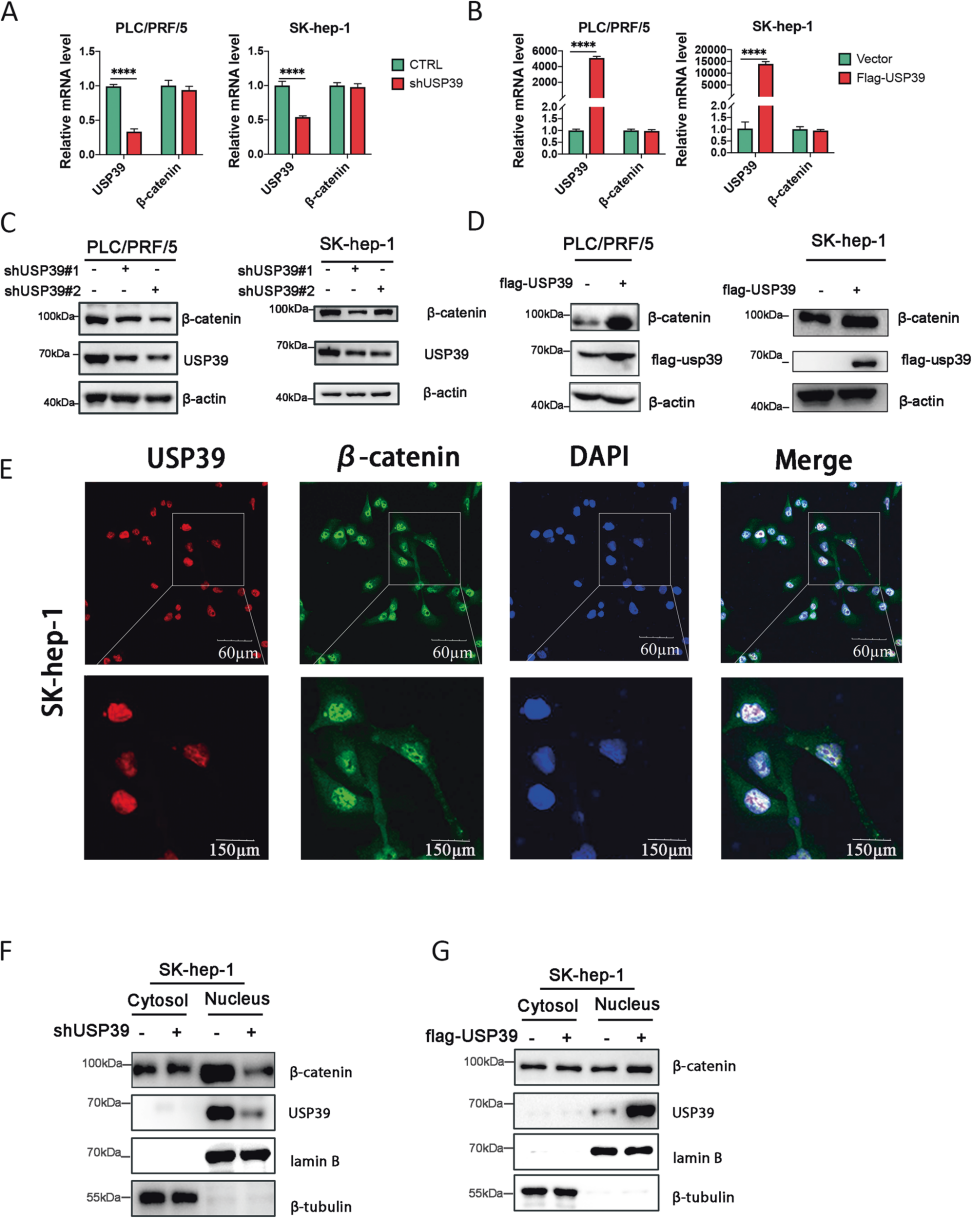
USP39 affected β-catenin expression and localization. RT-PCR analyzed the impact of knockdown (**A**) and overexpression (**B**) of USP39 on transcription levels of β-catenin. Western blotting detects the expression of β-catenin after cells were knockdown (**C**) and overexpression (**D**) of USP39. **E** Immunofluorescence staining assays of USP39 and β-catenin in HCC cells (SK-hep-1) observed by confocal microscopy (scale bar = 60 μm). Western blotting was performed to determine the distribution of β-catenin proteins in the nucleus and cytoplasm in SK-hep-1 after cells were knockdown (**F**) and overexpression (**G**) of USP39. All the data are representative of at least three independent experiments and presented as the means ± SD. (****p < 0.0001 v.s. control by Student’s *t*-test).

USP39 has the function of deubiquitination and regulating RNA splicing, and data above has proved USP39 increasing HCC progression through Wnt/β-catenin pathway. However, it is still unclear which role USP39 plays in the process. To determine how USP39 regulates β-catenin, qRT-PCR analyses of β-catenin mRNA level was performed in hepatoma cells with knockdown or overexpression of USP39. We found depletion (Fig. 2A) or overexpression (Fig. 2B) of USP39 made no change in β-catenin mRNA level. In contrast, western blotting revealed that knockdown of USP39 led to a reduction of β-catenin (Fig. 2C), and overexpression of USP39 increased β-catenin protein level (Fig. 2D) in HCC cells. Besides, immunofluorescence staining in SK-hep-1 showed that exogenous USP39 and β-catenin were co-localized in the nucleus (Fig. 2E). Detecting proteins separated from cytoplasmic and nuclear by western blotting showed that, knockdown of USP39 induced a reduction of nuclear β-catenin (Fig. 2F), whereas USP39 overexpression increased β-catenin level in the nucleus (Fig. 2G). Moreover, USP39 exerted its oncogenic properties by activating the relative genes of Wnt signals, including Axin, GSK3β, c-Myc and Cyclin D1 (Supplementary Fig. 1). These data suggested that USP39 affects the activity of the Wnt/β-catenin pathway.

### USP39 stabilizes β-catenin protein by deubiquitination

**Fig. 3:**
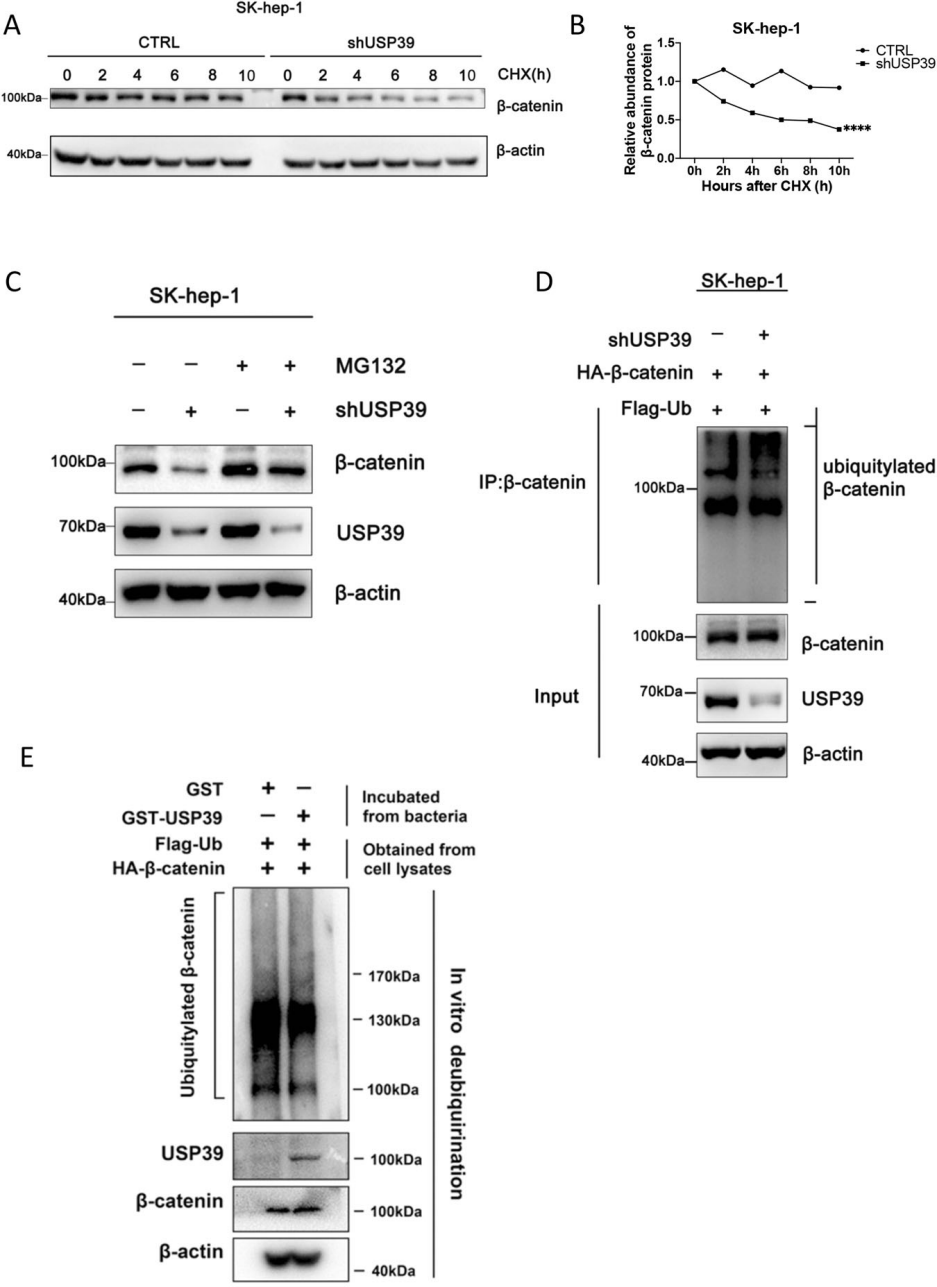
Loss of USP39 led to an increase in the ubiquitination of β-catenin. **A, B**. β-catenin protein level in SK-hep-1 cells under the downregulation of USP39 at the indicated times after CHX (0.2 mg/ml) addition. **C** The protein level of β-catenin in USP39 down-regulated SK-hep-1 cells treated with MG132 (20 μM), total protein was extracted and detected by Western blotting. **D** β-catenin ubiquitination in USP39 knockdown SK-hep-1 cells co-transfected with expression plasmids encoding HA-β-catenin and Flag-Ub. The transfected cells were treated with MG132 (20 μM for 4 h) prior to harvest. **E** β-catenin deubiquitination by USP39 in vitro using purified proteins. Whole-cell lysates (containing Flag-Ub and HA-β-catenin proteins) were incubated with purified GST-USP39 or GST-plasmid in vitro and blotted with USP39 or β-catenin antibodies. All the data are representative of at least three independent experiments and presented as the means ± SD. (****p < 0.0001 v.s. control by Student’s *t*-test).

On account of the fact that β-catenin protein level was decreased with no change on mRNA level, and USP39 is a deubiquitinating enzyme, we hypothesized whether USP39 de-ubiquitinate and stabilize β-catenin. To test this, we first proved that a combination between USP39 and β-catenin exists by immunoprecipitation in PLC/RCF/5 cells (Supplementary Fig. 2A). To further determine whether USP39 extends β-catenin’s half-life, β-catenin was monitored at different time points after treatment with protein inhibitor cycloheximide (CHX). In the absence of de novo protein synthesis, the half-life of endogenous β-catenin protein was shorter in USP39-depleted cells than in the control in SK-hep-1 cells (Fig. 3A, B). In contrast, overexpression of USP39 extended the half-life of β-catenin in PLC/RCF/5 cells (Supplementary Fig. 2B).

To further confirm whether the reduction of β-catenin is mediated through the ubiquitin-proteasome pathway, cells were treated with proteasome inhibitor MG132. As expected, downregulation of β-catenin was dramatically reversed in USP39 knockdown SK-hep-1 cells treated with MG132 (Fig. 3C). Furthermore, immunoprecipitation demonstrated that USP39 reduced the ubiquitination of β-catenin in SK-hep-1 cells (Fig. 3D). GST pull-down assay further demonstrated that USP39 could deubiquitylate β-catenin in vitro (Fig. 3E). Taken together, these data confirmed that USP39 directly interacted with β-catenin and regulated the ubiquitination of β-catenin to participate in the HCC progression.

### TRIM26 participates in USP39 regulating β-catenin and then affects HCC progression

**Fig. 4:**
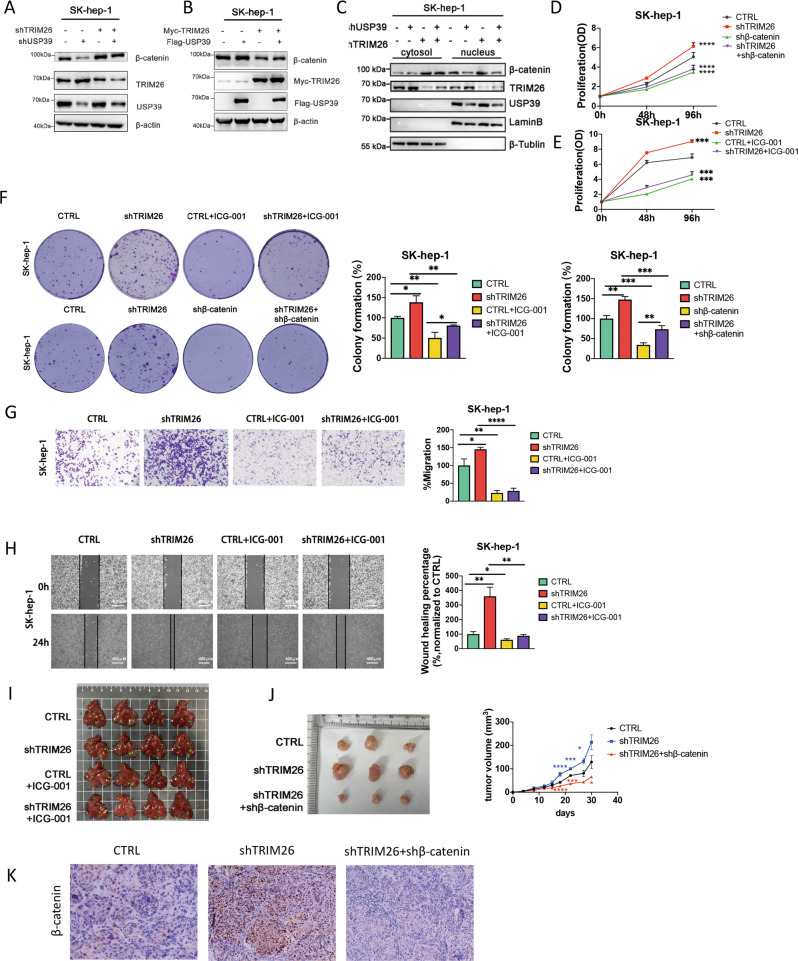
TRIM26 participates in USP39 regulating of β-catenin and involved in HCC progression. **A** SK-hep-1 cells were infected with either shUSP39, shTRIM26, or control plasmid, the protein level of β-catenin was determined by western blotting. **B** SK-hep-1 cells were transfected with Flag-USP39, Myc-TRIM26, or control plasmid, and western blotting was performed to detect β-catenin protein levels. **C** SK-hep-1 cells were infected with either shUSP39, shTRIM26 or both, proteins were separated from cytoplasmic and nuclear for western blotting. **D** The effect of silencing TRIM26 on the proliferation of SK-hep-1 cells was detected by MTT assay with or without β-catenin knockdown. **E** The effect of silencing TRIM26 on the proliferation of SK-hep-1 cells was examined by MTT assay in the presence or absence of Wnt pathway inhibitors ICG-001. **F** The colony counts were normalized to the control and expressed as a percentage, and the results are represented in the bar graph. **G** Representative images and Graphic representation of the migration capacities in TRIM26 and/or β-catenin knockdown SK-hep-1. **H** Representative images and Graphic representation of SK-hep-1 cell migration ability as shown by wound-healing assays. **I** Extrahepatic metastasis model was constructed in nude mice, Sk-hep-1 cells with knockdown of TRIM26 or treated with ICG-001, or shTRIM26 cells treated with ICG-001 were injected into nude mice through the caudal vein, and mice were sacrificed 40 days later to observe liver metastasis (*n* = 4). **J** The effect of TRIM26 and β-catenin in HCC cell proliferation in vivo was determined by xenograft assays. TRIM26 and β-catenin knockdown SK-hep-1 cells were respectively injected into flanks of BALB/c nude mice. After 30 days, tumors were isolated and photographed (*n* = 6), tumor volumes were calculated. **K** Representative images of IHC staining for β-catenin in tumor tissues from BALB/c nude mice. All the data are representative of at least three independent experiments and presented as the means ± SD (*p < 0.05; **p < 0.01; ***p < 0.001 v.s. control by one-way ANOVA/two-way ANOVA).

Considering β-catenin was proved to be a substrate of de-ubiquitinase USP39, and our previous research showed that TRIM26 balances the regulation of USP39 to its substrate, thus we assumed TRIM26 plays the same role during USP39 stabilizing β-catenin. To prove this assumption, we generated USP39 knockdown, TRIM26 knockdown and double-knockdown cell lines separately. Results showed that knockdown of USP39 led to an obvious decrease of β-catenin in HCC cells, whereas USP39 and TRIM26 double-knockdown significantly replenished β-catenin levels compared with USP39 deficit only (Fig. 4A). Furthermore, overexpression of USP39 increased β-catenin protein level, but co-overexpression of USP39 and TRIM26 counteracted the increase (Fig. 4B). Knockdown of USP39, TRIM26 or both, detecting proteins separated from cytoplasmic and nuclear by western blotting showed that USP39 and TRIM26 collectively affected β-catenin nuclear entry (Fig. 4C).

According to our previous findings, TRIM26 reduced the proliferation and migration of HCC cells. Here, to clarify whether TRIM26 regulates HCC progression via β-catenin, we generated TRIM26 and β-catenin knockdown HCC cell lines, and the knockdown efficiency was confirmed by qRT-PCR and WB (Supplementary Fig. 3). MTT experiments showed that the proliferation of HCC cells induced by TRIM26 knockdown was abolished when cells were treated with knockdown of β-catenin or ICG-001 (Fig. 4D, E). Consistently, colony formation assays revealed a significant increased clone number with knockdown of TRIM26, and this increase could be relieved by β-catenin inhibitor ICG-001 (Fig. 4F). Moreover, transwell (Fig. 4G) and wound-healing assay (Fig. 4H) showed that knockdown of TRIM26 increased HCC migration, as well, the effect was removed when Wnt signaling was inhibited by ICG-001.

To examine the effect of ICG-001 on CRC metastasis, we executed extrahepatic metastasis model in nude mice. Results showed that mouse injected with TRIM26 knock down SK-hep-1 cells showed more severe liver metastasis compared with the control group. The ICG-001 treatment significantly relieved metastatic outgrowth to the liver in both TRIM26 knock down and control group (Fig. 4I). To assess the effects of TRIM26 and β-catenin in the progression of HCC in vivo, TRIM26 and β-catenin knockdown SK-hep-1 cells were respectively injected into the flanks of BALB/c nude mice. Downregulation of TRIM26 expression in HCC cells significantly promotes tumor growth, with a greater volume of tumors. In contrast, tumors derived from shTRIM26 +shβ-catenin cells group were much smaller than the control group injecting with NC cells. (Fig. 4J). Immumohistochemical staining of β-catenin in tumors of shTRIM26 group showed a higher expression level (Fig. 4K). These results suggested that TRIM26 inhibits the proliferation and migration of HCC cells via the WNT signaling pathway both in vivo and in vitro.

### USP39 regulates TRIM26 pre-mRNA splicing

Our previous study indicated that de-ubiquitinase USP39 and E3 ligase TRIM26 function in an antagonistic but not a competitive pattern, and play key roles in controlling ZEB1 stability to determine the HCC progression [19]. However, weather a regulation exists between USP39 and TRIM26 is still not clear.

In this study we note that knockdown of USP39 induced an increase in TRIM26 protein level (Fig. 4A). To explore the mechanism behind this, we overexpressed and knockdown USP39, discovering that no effect on the half-life of TRIM26 when CHX inhibited new protein synthesis (Supplementary Fig. 4A, B). Moreover, ubiquitination level of TRIM26 was unaffected by knockdown of USP39 (Supplementary Fig. 4C). mRNA expression of TRIM26 in USP39 overexpression SK-hep-1 cell was down-regulated (Supplementary Fig. 4D).

**Fig. 5:**
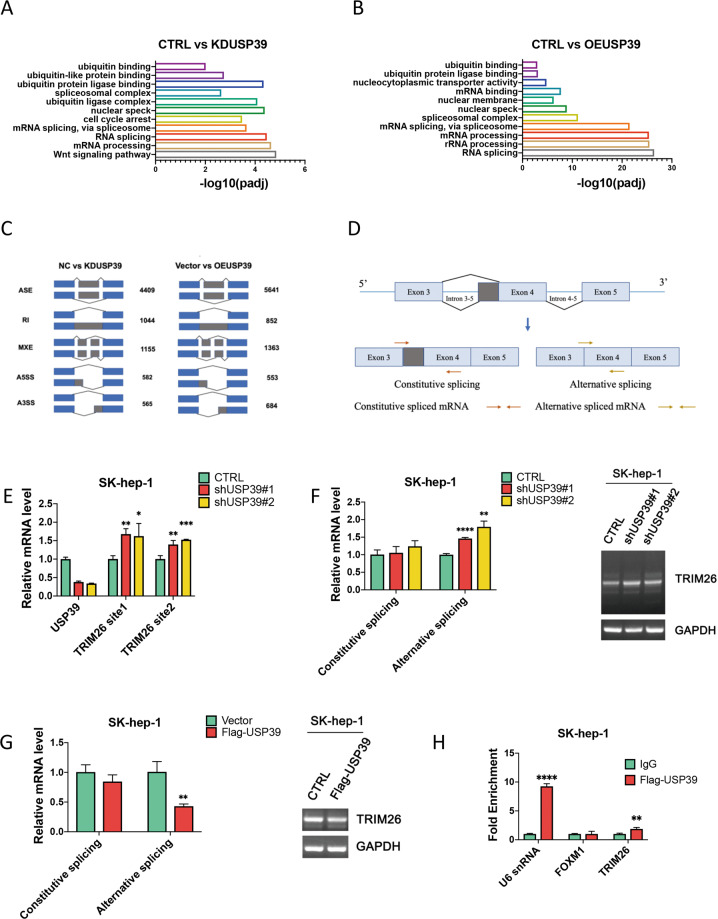
USP39 was involved in the splicing of TRIM26 pre-mRNA. Enriched GO analysis that presented different functions of differentially expressed genes (DEGs) from HCT116 cells knockdown (**A**) or expressing Flag-tagged USP39 (**B**) of USP39 **C** Number of significant differential splicing events identified following knockdown or overexpression of USP39 in human colon cancer cells using rMATS. (ASE alternative spliced exon, RI retained intron, MXE mutually exclusive exon, A5SS Alternative 5 splice site, A3SS Alternative 3 splice site.) **D** Schematic model representing the fourth exon region of TRIM26 selected for amplification experiments to detect constitutive and alternative spliced RNA transcripts using qRT-PCR. The specific primers designed are indicated by two pairs of arrows, indicating their approximate positions. **E** knockdown of USP39 in SK-hep-1, qRT-PCR was used to detect TRIM26 mRNA expression levels. **F** qRT-PCR was used to detect the expression of constitutive spliced and alternative spliced RNA transcripts in SK-hep-1 cells following the knockdown of USP39. **G** Overexpression of USP39 in SK-hep-1 cells, qRT-PCR was used to measure the levels of constitutive spliced and alternative spliced RNA transcripts. **H** RIP assay confirmed that USP39 binds to the mRNA of TRIM26. U6 snRNA and FoxM1 represented positive and negative control. All the data are representative of at least three independent experiments and presented as the means ± SD. (*p < 0.05; **p < 0.01; ***p < 0.001; ****p < 0.0001 v.s. control by one-way ANOVA or Student’s *t*-test).

USP39 has been reported to be involved in pre-mRNA splicing and maturation [33, 34]. Based on our previous study of RNA sequencing, enriched GO analysis indicated that differentially expressed genes in colon cancer cells HCT116 following USP39 knockdown (Fig. 5A) or overexpression (Fig. 5B) were mainly associated with the function of RNA splicing. Numbers of significant differential splicing events were identified following knockdown or overexpression of USP39 in human colon cancer cells using (Fig. 5C). Furthermore, rMATS analysis indicated that a significant change in exon 4 of TRIM26 mRNA exists, and we designed two pairs of primers near the exon 4 to detect the constitutive splicing mRNA and alternative splicing mRNA (Fig. 5D).

We first examined the effect of USP39 on the levels of TRIM26 mRNA by using qRT-PCR, discovering that knockdown of USP39 increased transcript level of TRIM26. (Fig. 5E). Using two pairs of primers, one for alternative splicing and the other for constitutive splicing of the TRIM26 mRNA, we confirmed that knockdown of USP39 increased alternative splicing of TRIM26 mRNA whereas constitutive splicing did not (Fig. 5F). Moreover, overexpression of USP39 reduced the alternative splicing of TRIM26 mRNA with no effect on the constitutive splicing (Fig.5G). RIP assay also confirmed that USP39 binds to the mRNA of TRIM26 (Fig. 5H). These data demonstrate that USP39 regulates TRIM26 pre-mRNA splicing and maturation, and led to decrease of TRIM26 protein level at least in part by suppressing splicing of its pre-mRNA.

### TRIM26 regulates β-catenin by ubiquitination

**Fig. 6:**
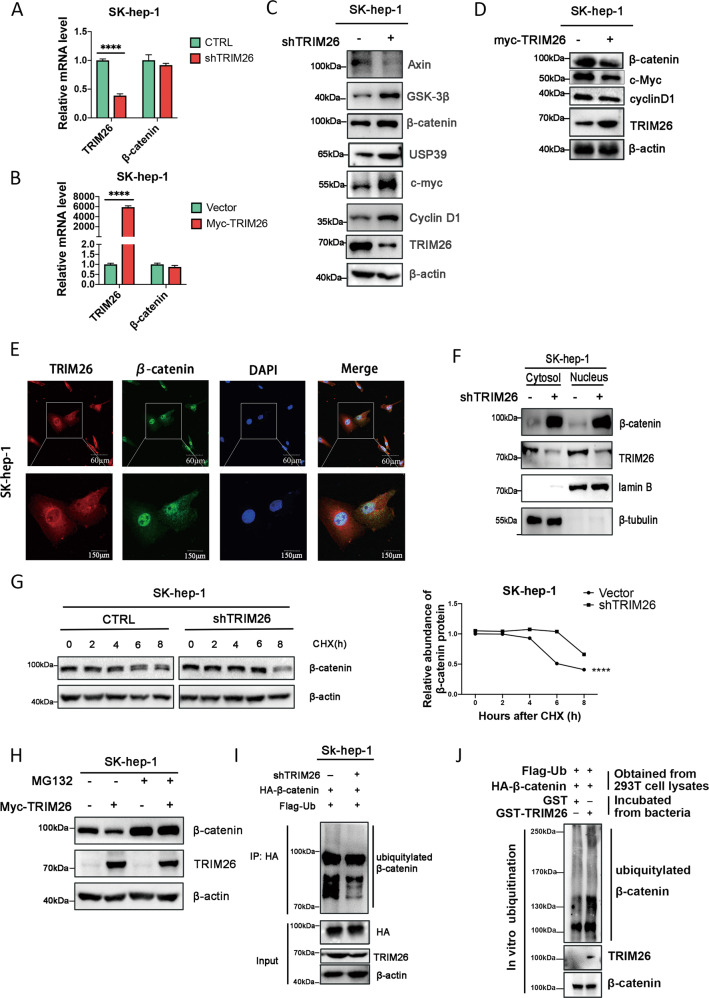
Knockdown of TRIM26 increased β-catenin protein levels. **A** The mRNA expression of β-catenin in shTRIM26-transfected SK-hep-1 cell was determined by qRT-PCR. **B** SK-hep-1 cells were transfected with plasmids overexpressing TRIM26 or control plasmid, and β-catenin mRNA levels were detected by qRT-PCR. **C** Effect of silencing TRIM26 on the expression levels of Wnt pathway-related proteins was detected by western blotting in SK-hep-1 cells. **D** The expression of Wnt pathway-related proteins were detected in overexpression TRIM26 SK- hep-1 cells by western blotting. **E** Immunofluorescence staining assays of TRIM26 and β-catenin in SK-hep-1 cells observed by confocal microscopy (scale bar = 60μm). **F** Western blotting was used to determine the effect of knockdown of USP39 on the intracellular distribution of β-catenin protein in SK-hep-1 cells. **G** SK-hep-1 cells with knockdown (or overexpression) of TRIM26 or control were treated with 0.2 μg/ml CHX, β-catenin protein level was detected at indicated time points by Western Blotting. **H** In TRIM26 overexpressing SK-hep-1 cells, MG132 was added to incubate for 24 h, and the protein level of β-catenin was detected by Western blotting. **I** Detection of ubiquitin status of β-catenin in SK-hep-1 cells with stable knockdown of TRIM26 by immunoprecipitation assay. **J** HEK-293T cells were transfected with HA-β-catenin and Flag-Ub, total protein was incubated with purified GST-TRIM26 or GST-Vector in vitro, and the ubiquitination of β-catenin was detected by western blotting. All the data are representative of at least three independent experiments and presented as the means ± SD. (****p < 0.0001 v.s. control by Student’s *t*-test).

Since TRIM26 possesses ubiquitin ligase activity [35, 36], we explored whether TRIM26 ubiquitinates and stabilizes β-catenin protein. qRT-PCR was performed and showed that β-catenin mRNA stayed at a level similar to control group no matter TRIM26 knockdown (Fig. 6A) or overexpression (Fig. 6B), excluding the possibility that β-catenin protein up-regulation resulted from transcription. Nevertheless, TRIM26-deficient HCC cells displayed a significant increase in the levels of β-catenin and Wnt-related proteins (Fig. 6C), and TRIM26 overexpression resulted in an opposite trend. (Fig. 6D). Double-immunofluorescent staining revealed a co-localization between TRIM26 and β-catenin in HCC cells (Fig 6E). To determine whether the cytoplasmic or nuclear β-catenin protein were decreased, we separated cytoplasmic and nuclear proteins and examined by Western blotting, proving that knockdown of TRIM26 increased β-catenin protein level both in nucleus and cytoplasm (Fig. 6F). Data above indicated that TRIM26 has no effect on β-catenin transcription level.

To examine whether TRIM26 affects the stability of β-catenin, CHX was performed to inhibit the synthesis of new proteins. Results showed that knockdown of TRIM26 prolonged the half-life of β-catenin (Fig. 6G). Consistently, overexpression of TRIM26 shortened the half-life of β-catenin (Supplementary Fig. 5A). We also observed that overexpression of TRIM26 reduced the level of β-catenin protein, which was blocked by MG132 through its inhibition to proteasome pathway (Fig. 6H). Finally, immunoprecipitation experiments confirmed that TRIM26 knockdown reduced the ubiquitination level of β-catenin (Fig. 6I). On the contrary, TRIM26 overexpression promoted the ubiquitination of β-catenin (Supplementary Fig. 5B). Furthermore, GST pull-down experiment proved that TRIM26 increased the levels of β-catenin ubiquitination directly (Fig. 6J). Altogether, these data strongly supported that USP39 deubiquitinates β-catenin, and suppressed TRIM26 pre-mRNA maturation, further influenced its protein level and decreased its ubiquitination to β-catenin, which plays a key role in promoting HCC progression.

## Discussion

Current studies on cancer have shown that ubiquitination-deubiquitination signaling pathway plays a key role in the regulation of cell homeostasis, especially in the occurrence and development of tumors, and its dysregulation will lead to tumor deterioration [37]. In the past, some limitations were in the understanding of USP39, which was once believed that USP39 did not have deubiquitination activity because of three amino acid residues mutations in its DUB domain critical for deubiquitination activity [38]. This also makes the USP39 somewhat controversial in its function. Our previous studies showed that the deubiquitination enzyme USP39 and the E3 ubiquitin ligase TRIM26 balance the ubiquitination level of ZEB1, thereby determining the progression of hepatocellular carcinoma. This finding firstly provided the indication of the deubiquitination activity of USP39 and further demonstrated its critical role in HCC progression. Moreover, studies showed that E3 ubiquitin ligases and deubiquitination enzymes form a potential target family for enhanced antitumor therapy [39]. However, whether USP39 affects HCC progression by regulating the ubiquitination level of other substrates or by regulating pre-mRNA maturation is still not clear.

Evidence showed that dysregulation of Wnt signaling contributes to the progression of hepatocellular carcinoma [40]. Liu et al. reported that Shc3 inhibits its ubiquitin-degrading pathway by interacting with β-catenin to promote HCC cells stemness and drug resistance [41]. β-catenin destruction complex and β-catenin degradation regulated by the ubiquitin-proteasome pathway are the core mechanisms regulating intracellular β-catenin levels [42]. This suggested that USP39 may affect the progression of HCC by regulating the protein level of β-catenin. In our further study, the ubiquitination level of β-catenin was proved to be affected by USP39, indicating that β-catenin was another substrate for USP39 deubiquitination.

As an important regulator of RNA splicing, USP39 participated in mRNA splicing in the process of cell proliferation [43]. In addition, numerous studies showed that USP39 was involved in cancer progression in various human tumor types and functions as a splicing factor. Loss of USP39 leads to aberrant RB1 mRNA splicing, which may lead to increased expression of its target E2F4, a key regulator known to have oncogenic activity when overexpressed [44]. USP39 regulates mTORC2 activity by selectively enhancing the splicing and maturation of Rictor mRNA to promote ESCC [45].

Although our previous study found that knockdown of USP39 could increase the protein level of TRIM26, no further exploration was performed to the mechanism of USP39 regulating TRIM26. In this study, our RNA sequencing data of USP39 overexpression in HCT116 cells revealed a significant difference in alternative splicing events in exon 4 of TRIM26 compared to controls. We confirmed that USP39 deletion increased the alternate splicing transcript of TRIM26 without significant changes in its constitutive splicing. More importantly, RNA immunoprecipitation showed that USP39 is bound to TRIM26 mRNA. We demonstrated for the first time that USP39 regulates TRIM26 pre-mRNA splicing and maturation, and the decrease in TRIM26 protein level caused by USP39, was at least in part by inhibiting the splicing of its pre-mRNA.

Considering the relationship between USP39 and TRIM26 in our previous study, we also wondered whether USP39 and TRIM26 could balance the ubiquitination level of β-catenin. Double knockdown of TRIM26 and USP39 eliminated the reduction of β-catenin caused by USP39 silencing. According to our previous data, knockdown of TRIM26 promoted HCC proliferation, colony formation, migration, and invasion. We double knockdown TRIM26 and β-catenin or ICG-001 treated in HCC cells, detecting that β-catenin knockdown and ICG-001 knockdown reduced the proliferation, colony formation, migration and invasion of HCC compared to TRIM26 knockdown only. Based on the above studies, we demonstrated that TRIM26 was partially involved in the regulation of β-catenin by USP39 and involved in the progression of HCC.

Because of the E3 ubiquitin ligase activity of TRIM26, we considered TRIM26 has the function of promoting β-catenin ubiquitination and degradation. Immunofluorescence assay showed co-localization of TRIM26 and β-catenin, and immunoprecipitation assay confirmed that TRIM26 is directly bound to β-catenin. Finally, by immunoprecipitation and GST pull-down assay, we demonstrated that TRIM26 ubiquitinates β-catenin in HCC cells.

Taken together, our experimental data support the mechanism by which USP39 regulates HCC cell proliferation and migration through the TRIM26/β-catenin axis. The elucidation of this mechanism will provide new clues for the development of hepatocellular carcinoma drugs.

## Supplementary information

supplementary figure 1

supplementary figure 2

supplementary figure 3

supplementary figure 4

supplementary figure 5

supplementary figure legends

Supplementary Table 1

Original Data File

aj-checklist

## Data Availability

All datasets generated and analyzed during this study are included in this published article and its Supplementary Information files. Additional data are available from the corresponding author upon reasonable request.
