# Supplementary figures and images for: USP39 stabilizes β-catenin by deubiquitination and suppressing E3 ligase TRIM26 pre-mRNA maturation to promote HCC progression
Source: Cell Death Dis. 2023 Jan 27;14(1):63. doi: 10.1038/s41419-023-05593-7 (PMC9883245; doi:10.1038/s41419-023-05593-7)

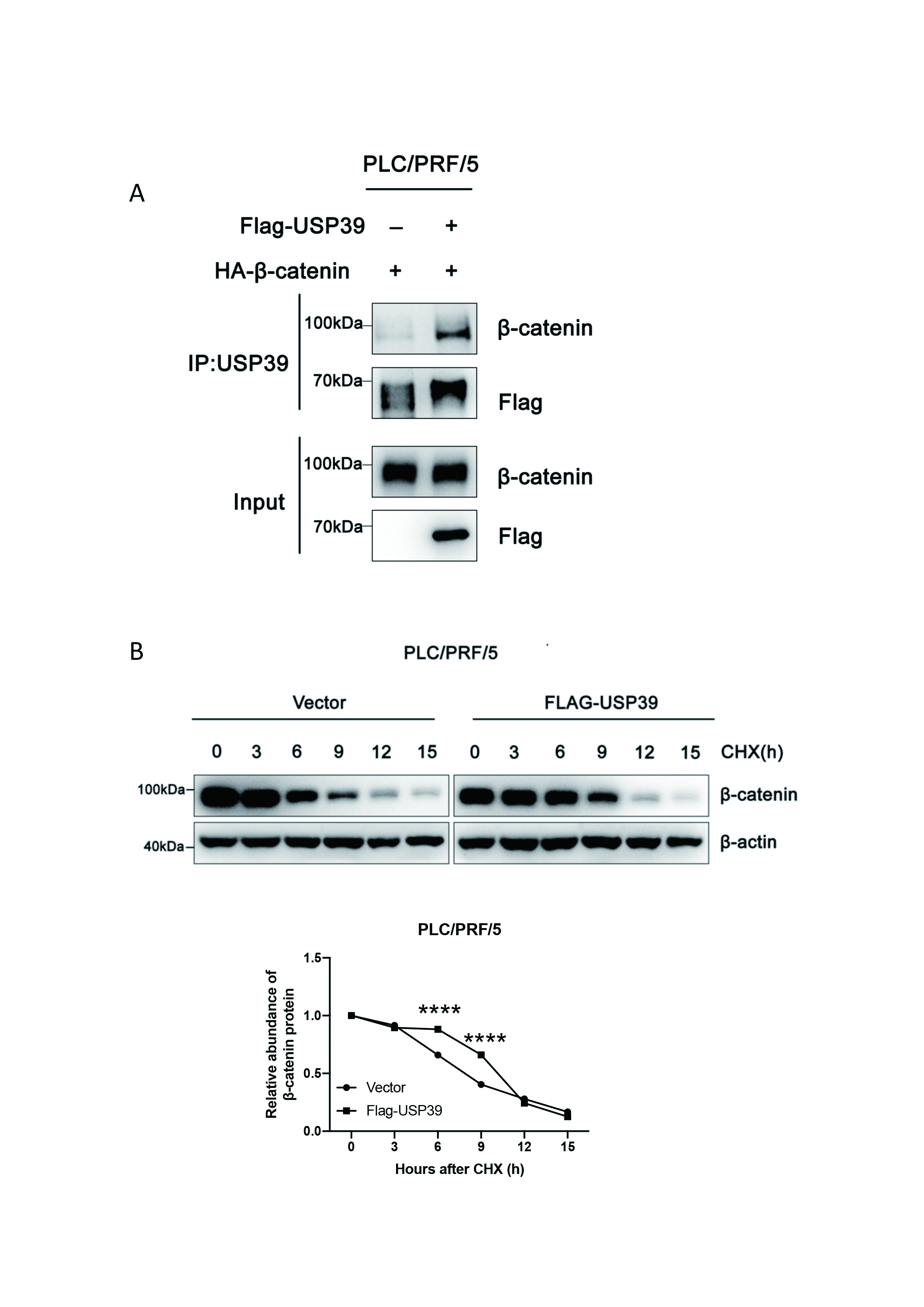

Supplement: Supplementary file 2 — supplementary figure 2 [file 41419_2023_5593_MOESM2_ESM.tif]
