# Supplementary material for: USP39 stabilizes β-catenin by deubiquitination and suppressing E3 ligase TRIM26 pre-mRNA maturation to promote HCC progression
Source: Cell Death Dis. 2023 Jan 27;14(1):63. doi: 10.1038/s41419-023-05593-7 (PMC9883245; doi:10.1038/s41419-023-05593-7)
Supplement: Supplementary file 6 — supplementary figure legends [file 41419_2023_5593_MOESM6_ESM.docx]

**Fig.S1**

**A.** Western blot detects the expression of Wnt signaling-related proteins after cells were knockdown USP39.

**Fig.S2**

**A**. Interaction of exogenous USP39 and β-catenin in PLC/PRF/5 cell. HA-flag antibody was immunoprecipitated, and USP39 bound to β-catenin was determined using immunoblotting (IB) with an anti-β-catenin antibody. **B.** β-catenin protein level in PLC/PRF/5 cells by overexpression of USP39 at the indicated times after CHX (0.2mg/ml) addition. All the data are representative of at least three independent experiments and presented as the means ± SD. (*****p* < 0.0001 *v. s.* control by Student’s t-test).

**Fig.S3**

**A-B** The expression of TRIM26 mRNA (**A**) and β-catenin mRNA (**B**) was analyzed by qRT-PCR in SK-hep-1 cells. **C.** Western blotting confirmed the expression of TRIM26 after cells were knocked down in SK-hep-1 cells. **D.** Western blotting confirmed the expression of β-catenin after cells were knocked down in SK-hep-1 cells. All the data are representative of at least three independent experiments and presented as the means ± SD. (*** *p* < 0.001 *v. s.* control by Student’s t-test).

**Fig.S4**

**A.** TRIM26 protein level in SK-hep-1 cells under the down-regulation of USP39 at the indicated times after CHX (0.2mg/ml) addition. **B.** Level of TRIM26 in USP39 overexpression PLC/PRF/5 cells at the indicated times after CHX (0.2mg/ml) addition. **C.** The ubiquitination of TRIM26 in USP39 knockdown SK-hep-1 cells co-transfected with expression plasmids encoding Myc-TRIM26 and Flag-Ub. The transfected cells were treated with MG132 (20 μM for 4 h) prior to harvest. **D.** The mRNA expression of TRIM26 in USP39 overexpression SK-hep-1 cell was determined by qRT-PCR. All the data are representative of at least three independent experiments and presented as the means ± SD. (**p* < 0.05 *v. s.* control by Student’s t-test).

**Fig.S5**

**A.** Levels of β-catenin in TRIM26 overexpression PLC/PRF/5 cells at the indicated times after CHX (0.2mg/ml) addition. **B.** Interaction of exogenous TRIM26 and β-catenin in PLC/PRF/5 cells. HA-flag antibody was immunoprecipitated, and TRIM26 bound to β-catenin was determined using immunoblotting (IB) with β-catenin antibody. All the data are representative of at least three independent experiments and presented as the means ± SD. (*****p* < 0.0001 *v. s.* control by Student’s t-test).
