# Supplementary material for: USP39 stabilizes β-catenin by deubiquitination and suppressing E3 ligase TRIM26 pre-mRNA maturation to promote HCC progression
Source: Cell Death Dis. 2023 Jan 27;14(1):63. doi: 10.1038/s41419-023-05593-7 (PMC9883245; doi:10.1038/s41419-023-05593-7)
Supplement: Supplementary file 7 — Supplementary Table 1 [file 41419_2023_5593_MOESM7_ESM.docx]

**Table 1**

| **Primer** | Sequence 5'-3' |
| --- | --- |
| human GAPDH-F | CTGGGCTACACTGAGCACC |
| human GAPDH-R | AAGTGGTCGTTGAGGGCAATG |
| human CTNNB1 F | TACCTCCCAAGTCCTGTATGAG |
| human CTNNB1 R | TGAGCAGCATCAAACTGTGTAG |
| human TRIM26 F | GAAGCTGCTGTGCGTGATG |
| human TRIM26 R | GCTTCTCCCTTTGCCTGGAAG |
| human USP39 F | TTGGAAGAGGCGAGATAA |
| human USP39 R | AGGAGCATCAATCATCATC |
| human U6 snRNA F（RIP） | CTCGCTTCGGCAGCACA |
| human U6 snRNA R（RIP） | AACGCTTCACGAATTTGCGT |
| human TRIM26 F（RIP） | CAAGTCCTCCAGGAGCTGGT |
| human TRIM26 R（RIP） | GGTCTCCGTTCACTGGTGAG |
| human FOXM1 F（RIP） | TTCTCTCCCACAATGCCTGG |
| human FOXM1 R（RIP） | CTCTGTCCACTAAAGCCATGC |
| human TRIM26 F（[constitutive](javascript:;) [splicing](javascript:;)） | AAGTCCTCCAGGAGCTGGT |
| human TRIM26 R（[constitutive](javascript:;) [splicing](javascript:;)） | GACGTGGCCATGGTATCCTT |
| human TRIM26 F（alternative splicing） | GTCCTCCAGGAGAAGTCC |
| human TRIM26 R（alternative splicing） | CAGAAGACGTGGCCACAGT |
