# Supplementary material for: USP39 stabilizes β-catenin by deubiquitination and suppressing E3 ligase TRIM26 pre-mRNA maturation to promote HCC progression
Source: Cell Death Dis. 2023 Jan 27;14(1):63. doi: 10.1038/s41419-023-05593-7 (PMC9883245; doi:10.1038/s41419-023-05593-7)

Fig1B

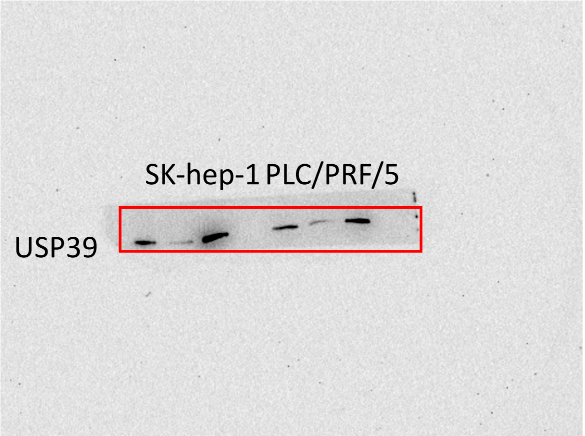

SK-hep-1 PLC/PRF/5
USP39

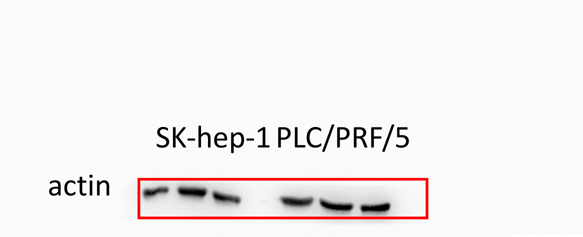

SK-hep-1 PLC/PRF/5
actin

Fig2C

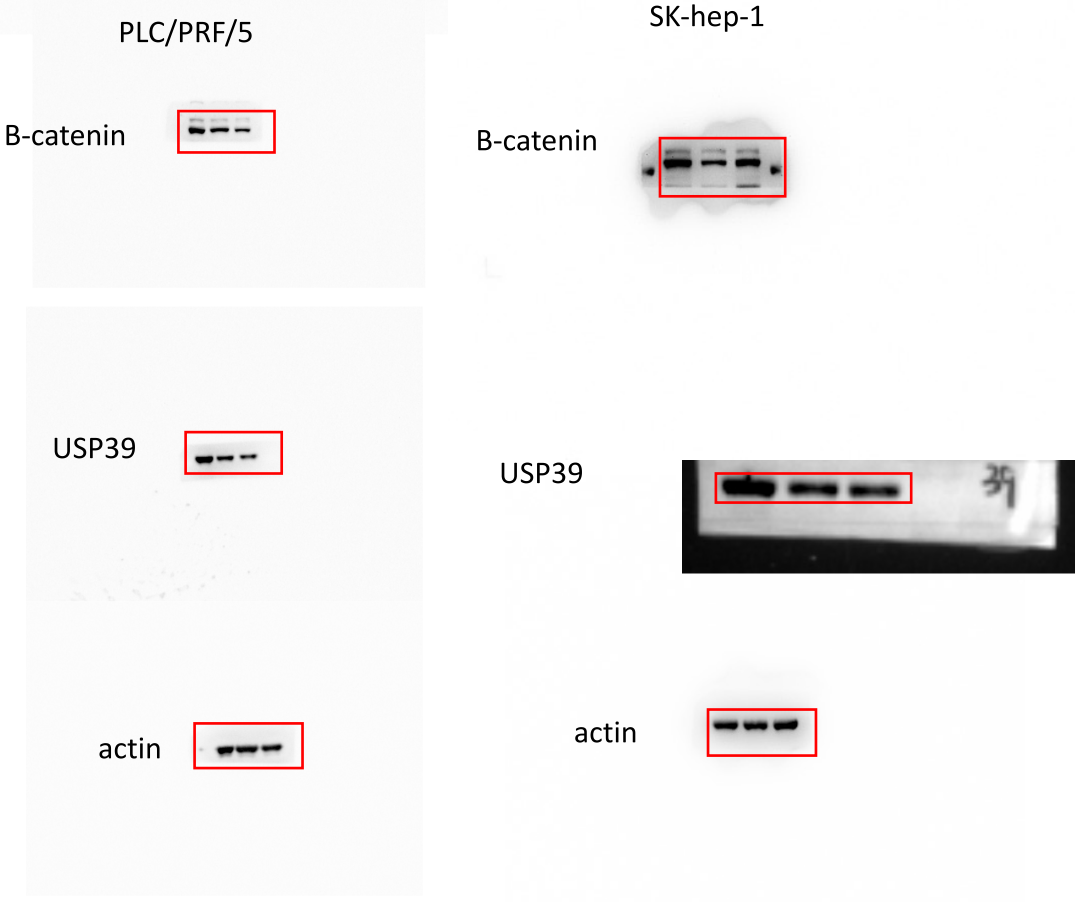

PLC/PRF/5
SK-hep-1
B-catenin
B-catenin
USP39
USP39
actin
actin

Fig2D

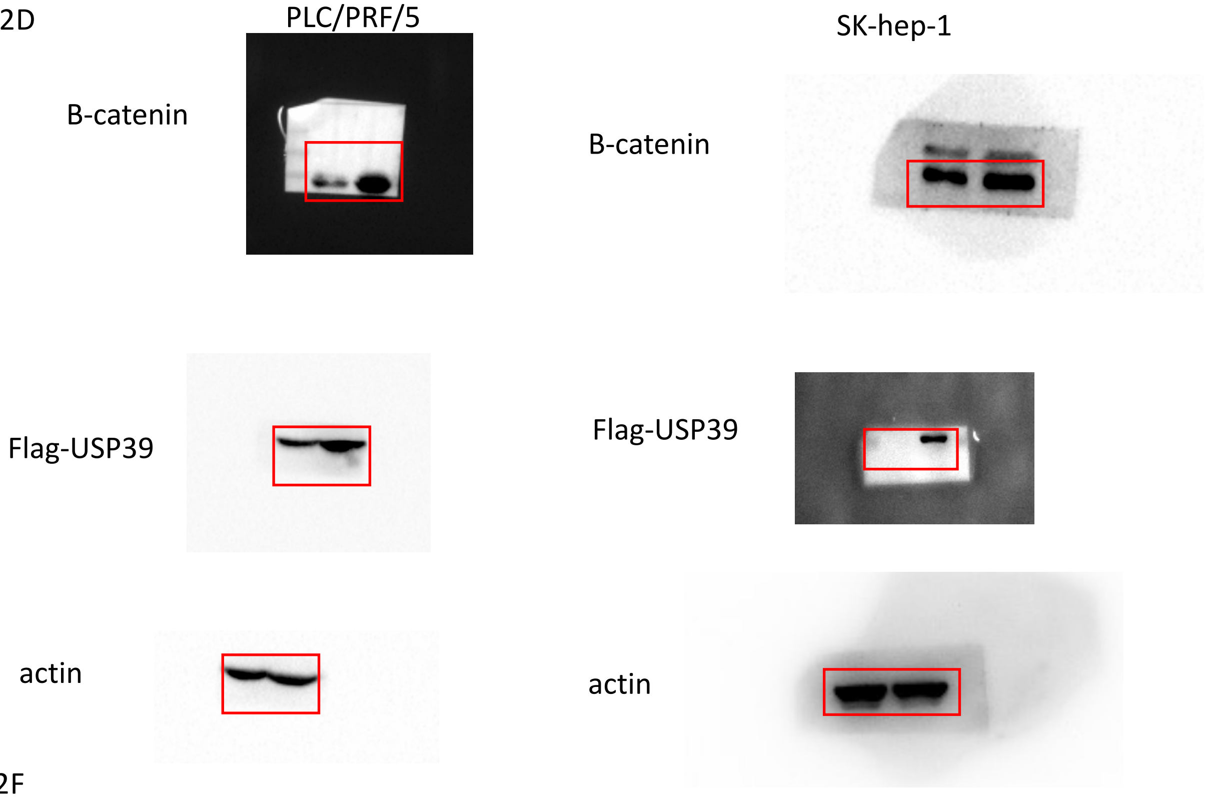

Fig2F

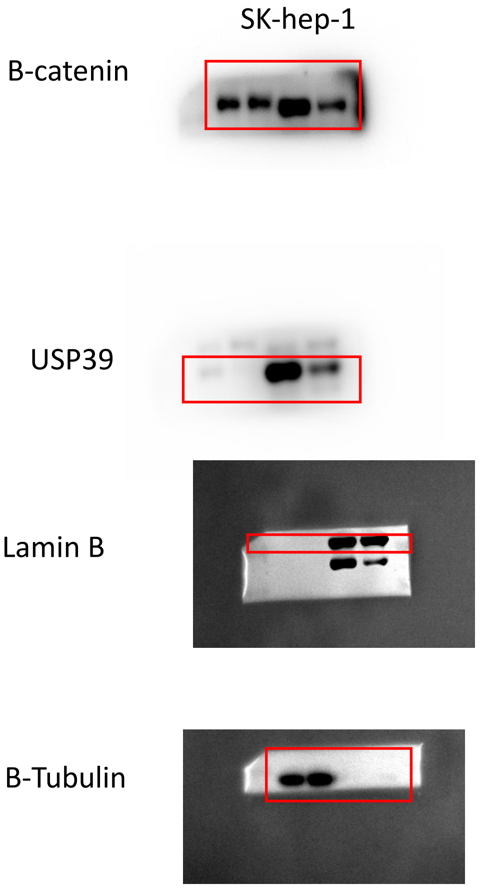

Fig2G

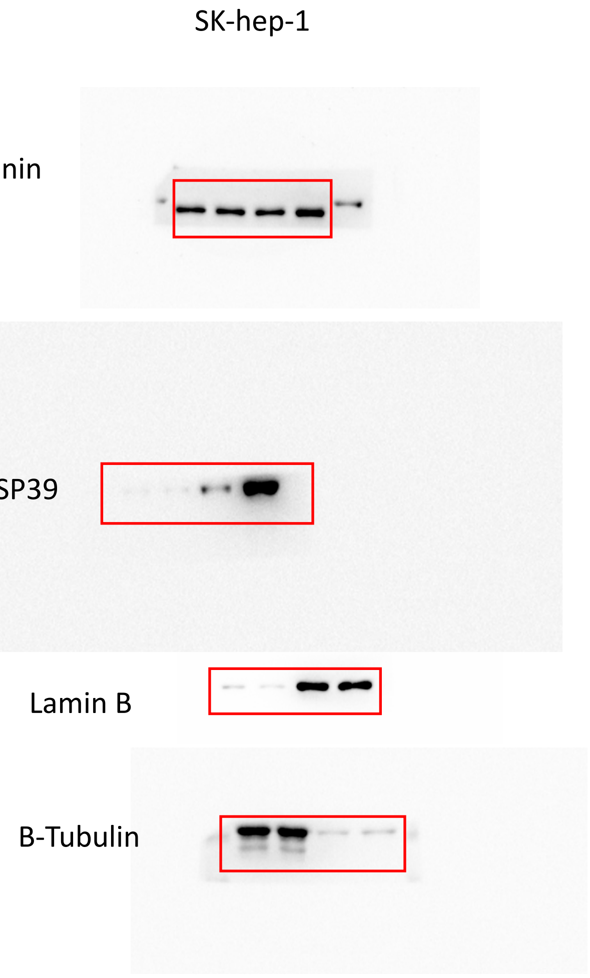

Fig3A

SK-hep-1

b-catenin

B-actin

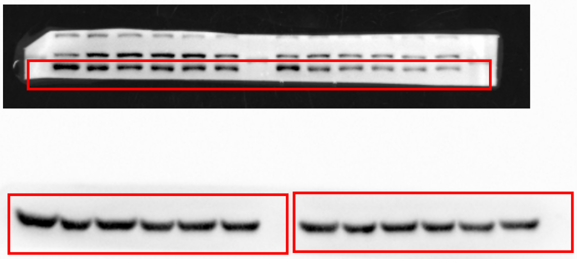

Fig3C

SK-hep-1

b-catenin

USP39

B-actin

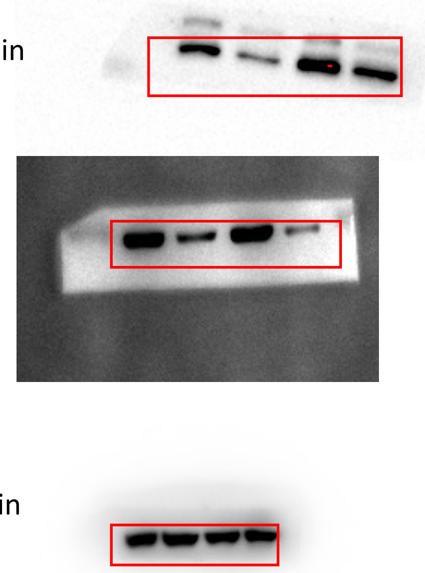

Fig3D

Ubiquitylated b-catenin

b-catenin

USP39

B-actin

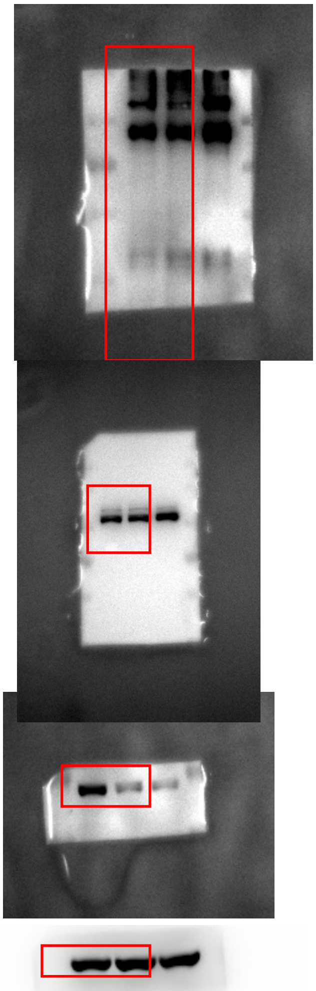

fig3E

Ubiquitylated b-catenin

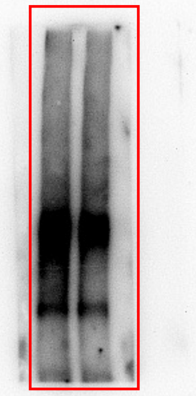

USP39

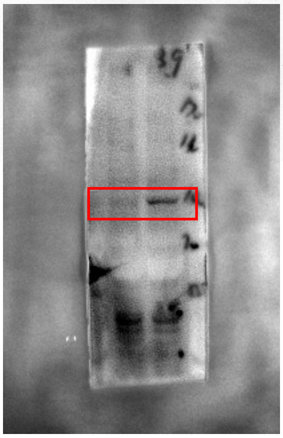

b-catenin

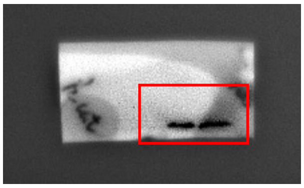

B-actin

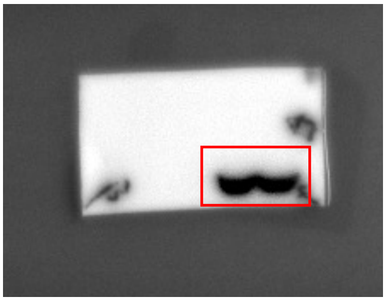

fig4A

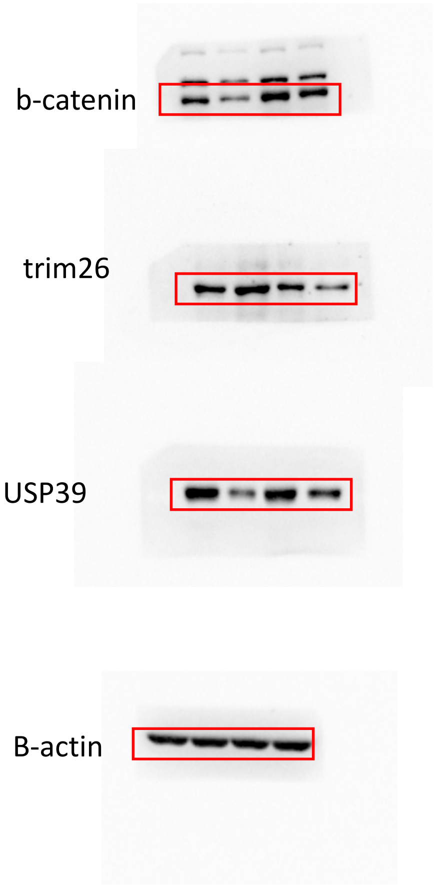

fig4B

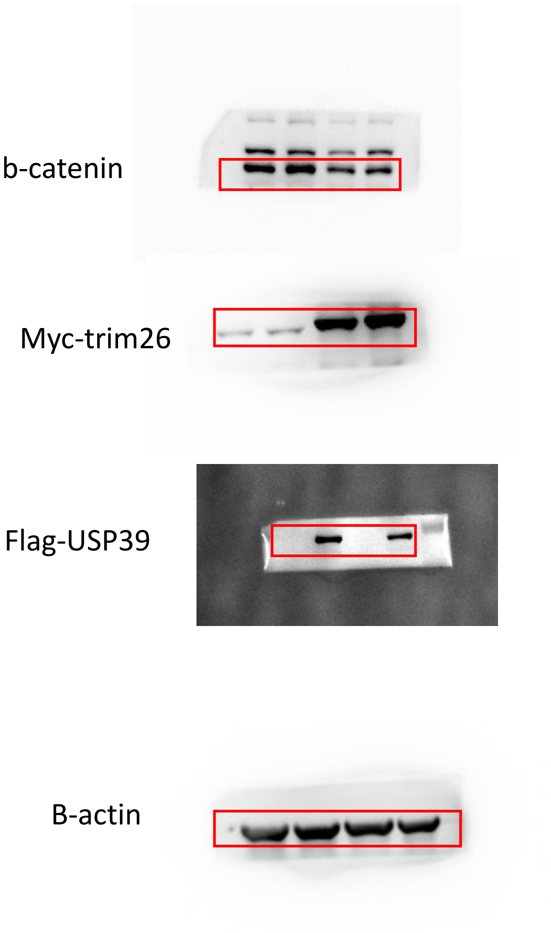

fig4C

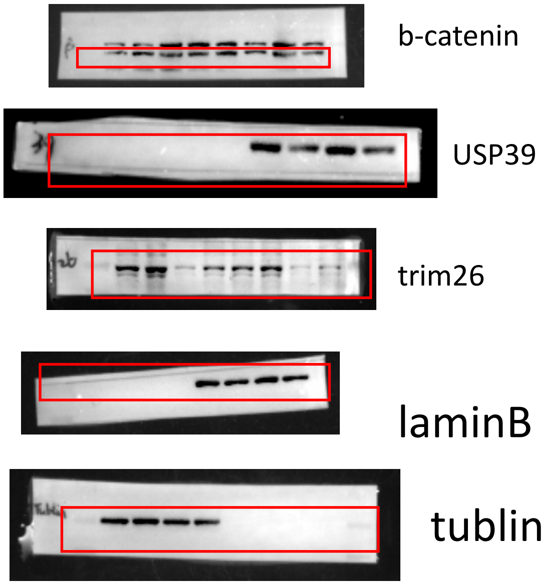

fig6C

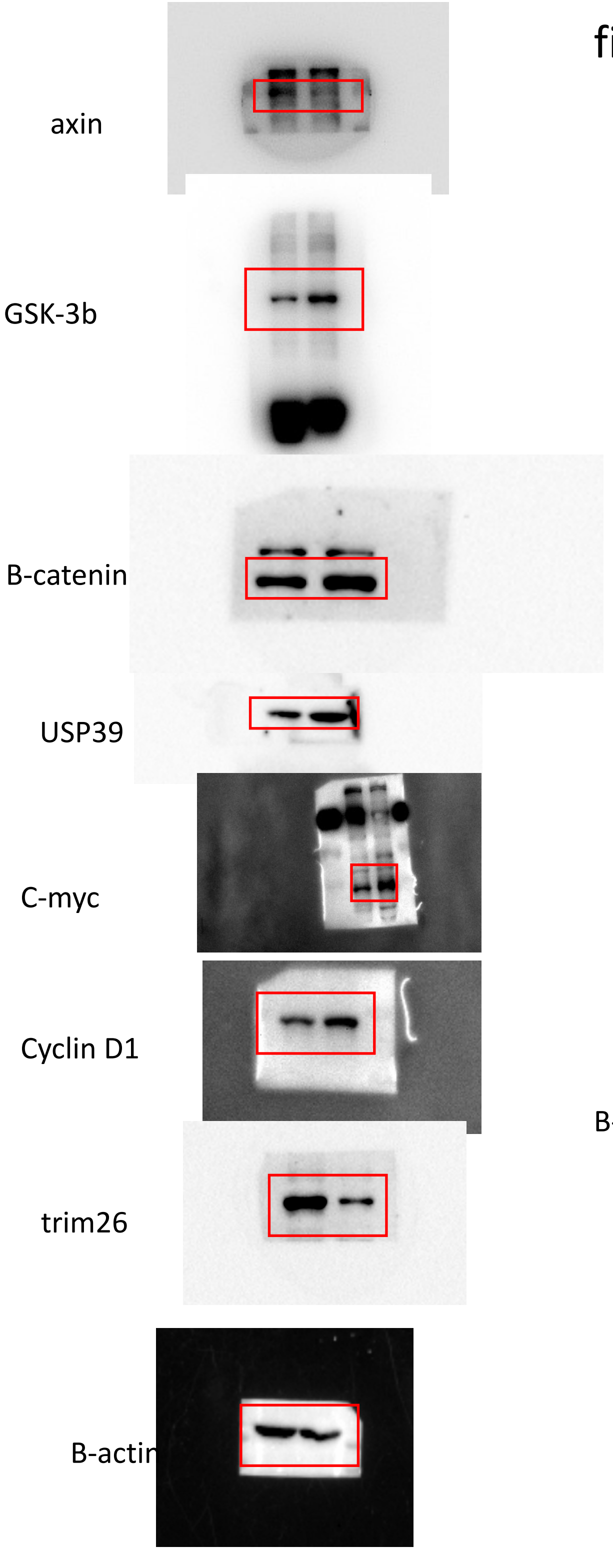
axin
GSK-3b
B-catenin
USP39
C-myc
Cyclin D1
trim26
B-actin

fig6D

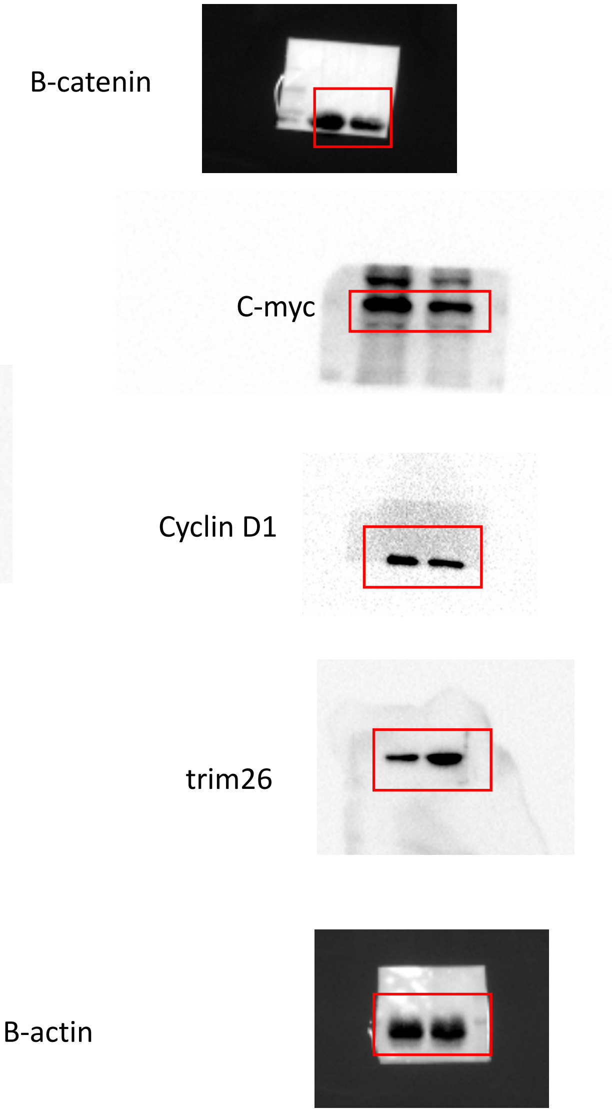
B-catenin
C-myc
Cyclin D1
trim26
B-actin

Fig6F

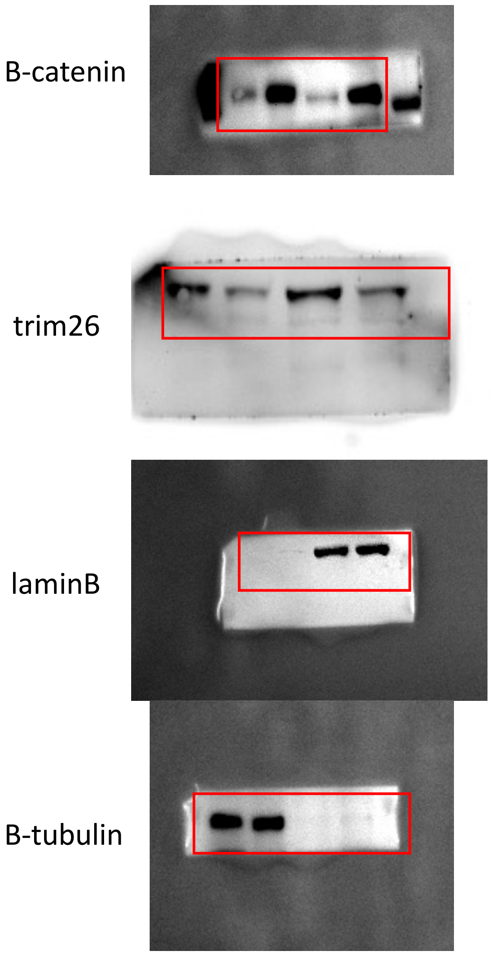

Fig6G

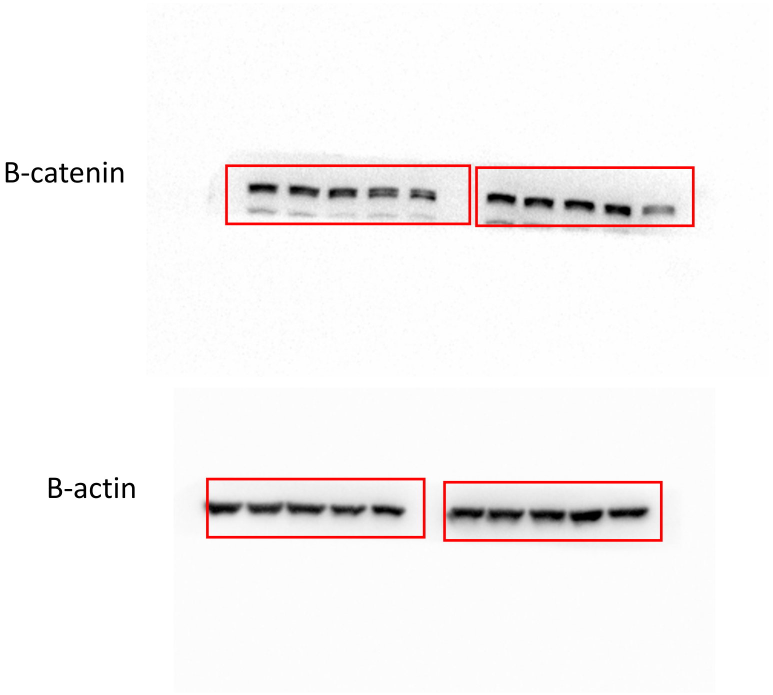

Fig6H

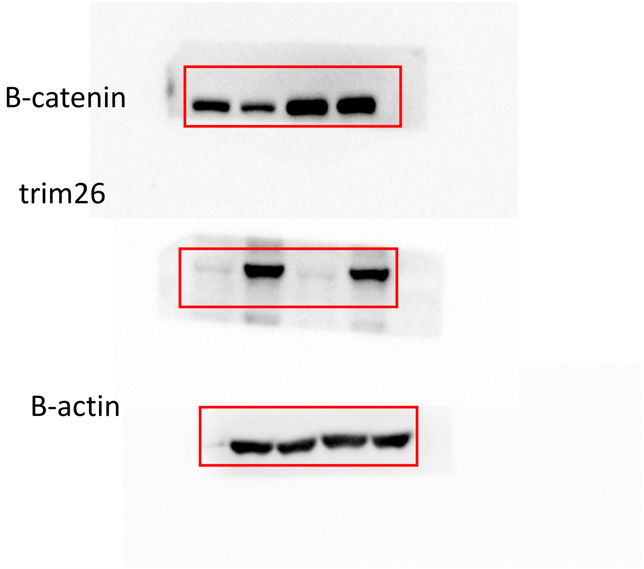

Fig6I

Ubiquitylated B-catenin

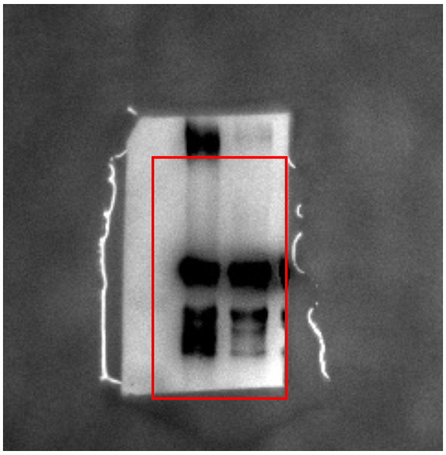

B-catenin

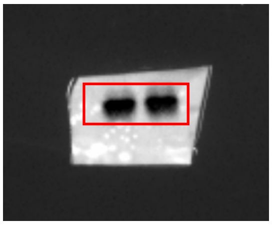

trim26

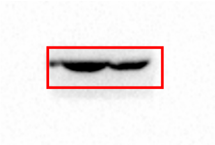

actin

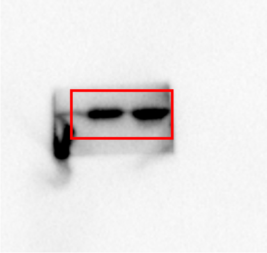

Fig6J

Ubiquitylated B-catenin

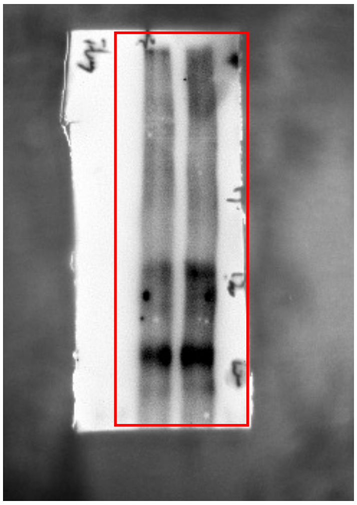

trim26

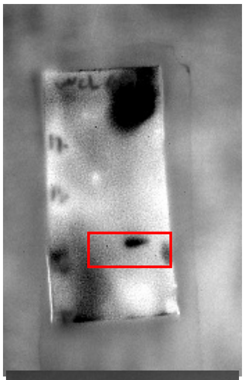

B-catenin

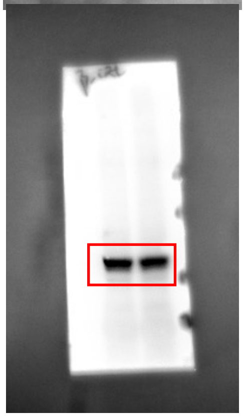

Fig S1

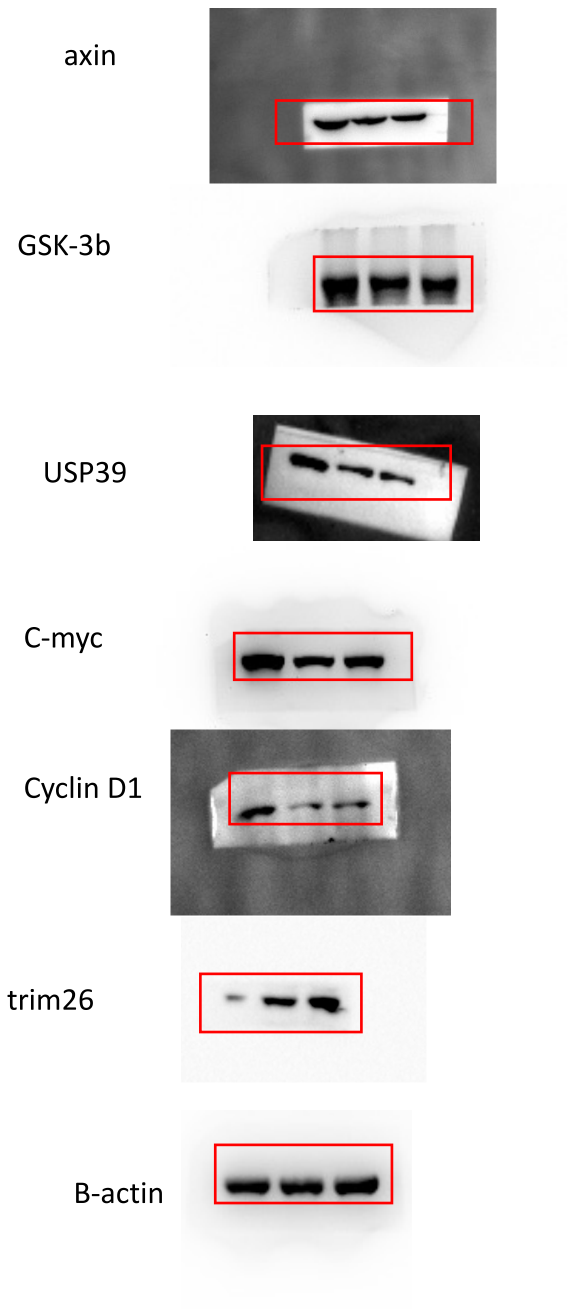

figS2A

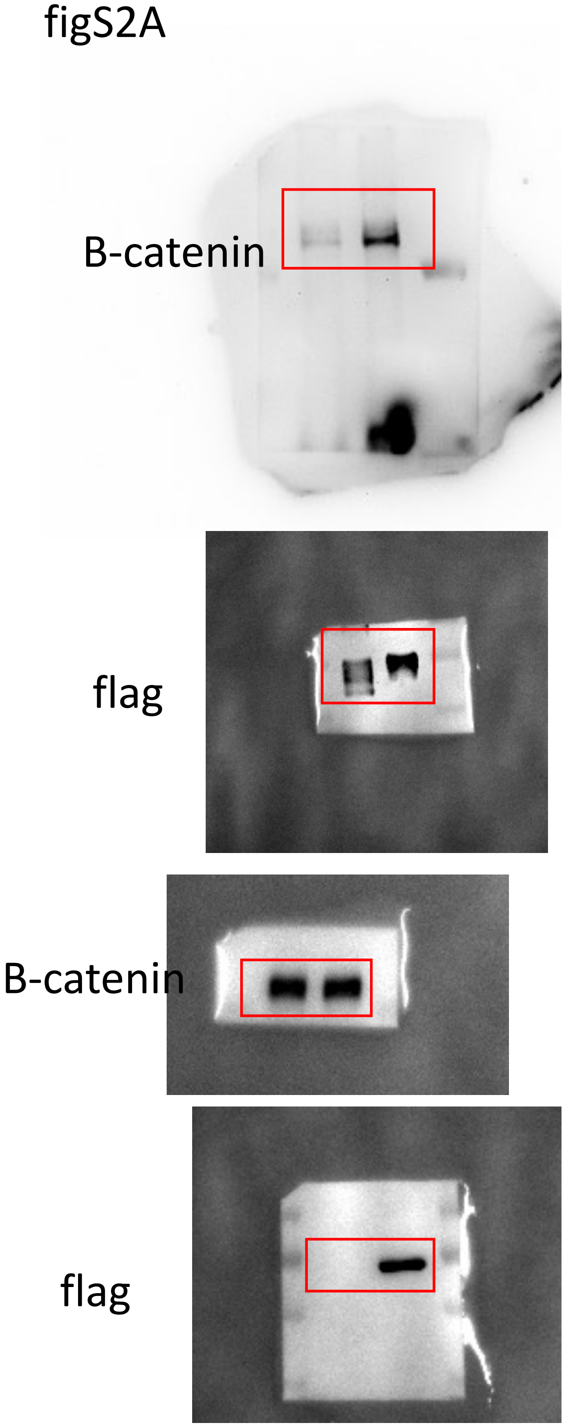

figS2B

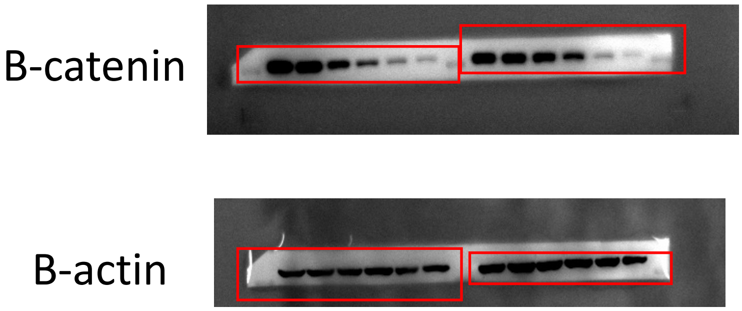

FigS3C

SK-hep-1

trim26

B-actin

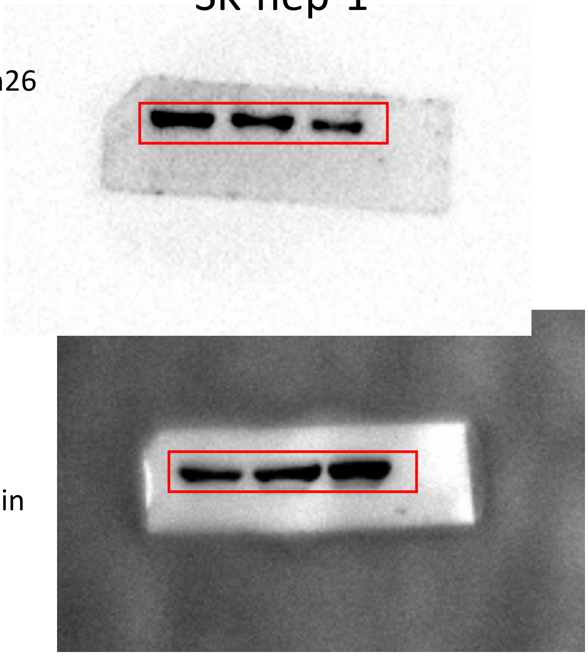

FigS3D

SK-hep-1

B-catenin

B-actin

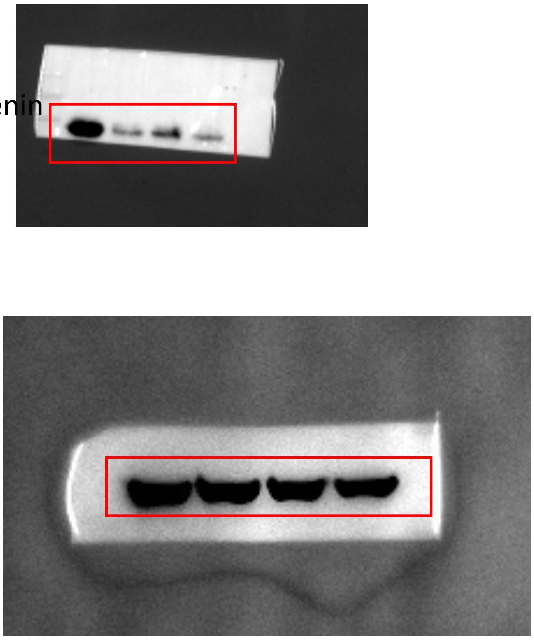

FigS4A

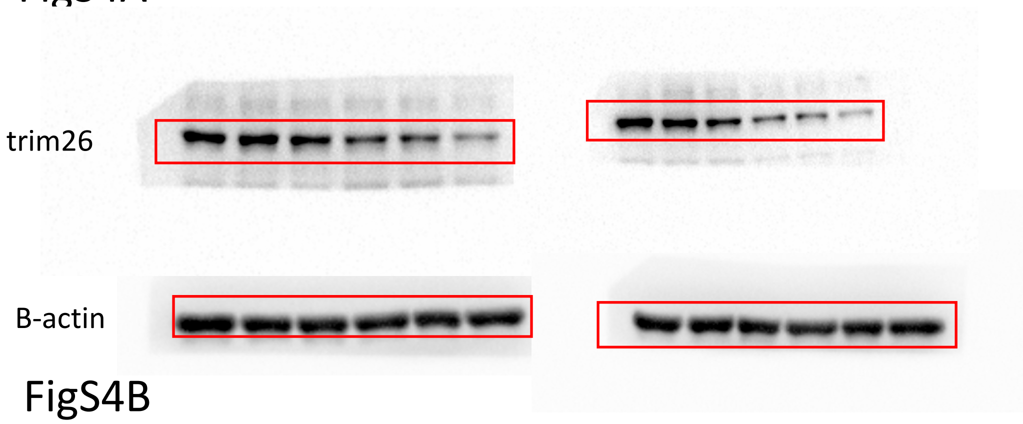

FigS4B

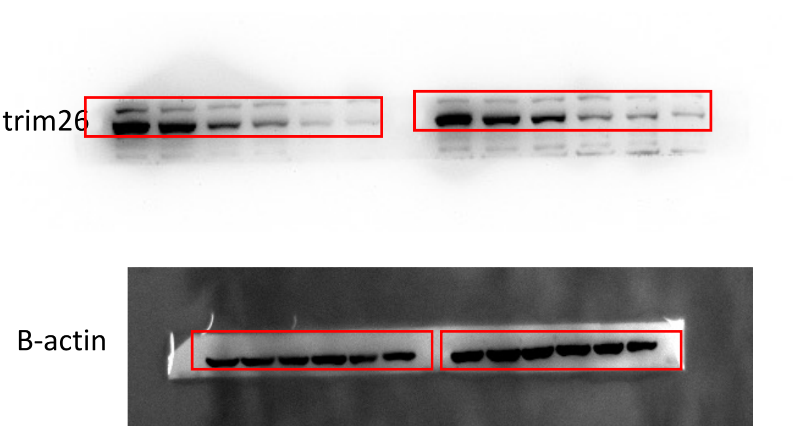

FigS4C

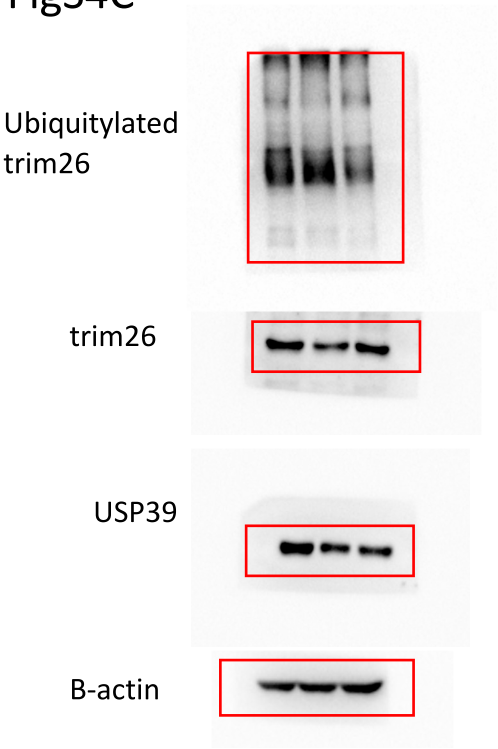

FigS5A

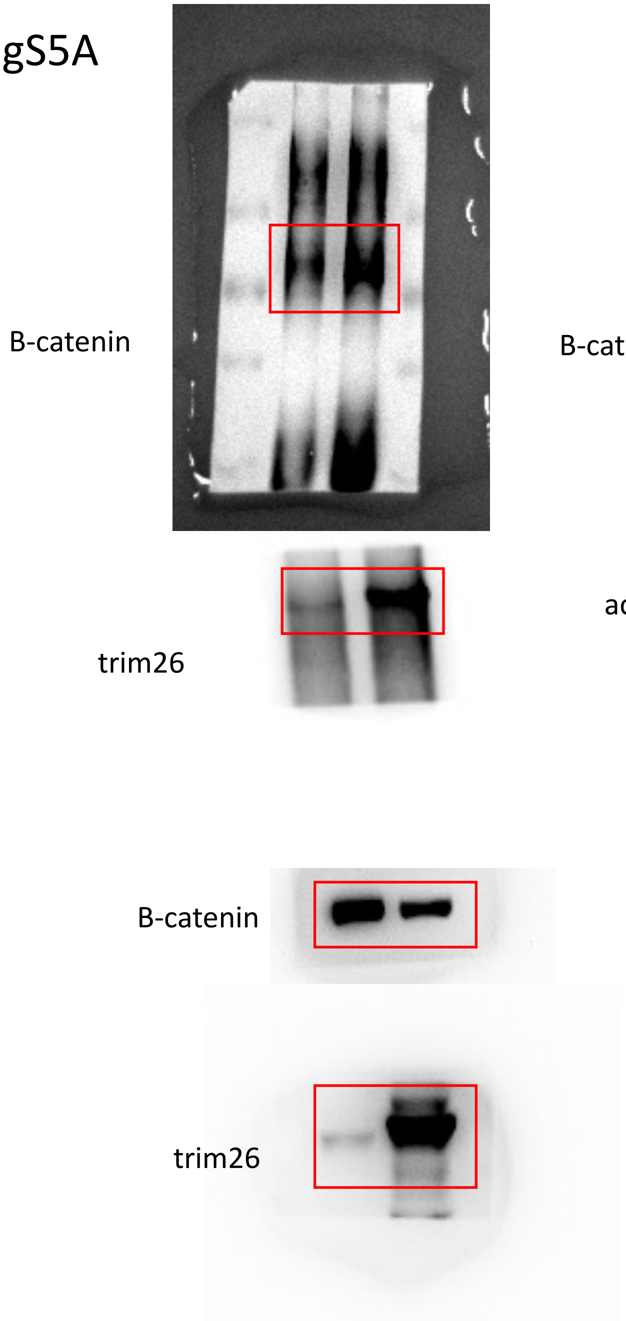

FigS5B

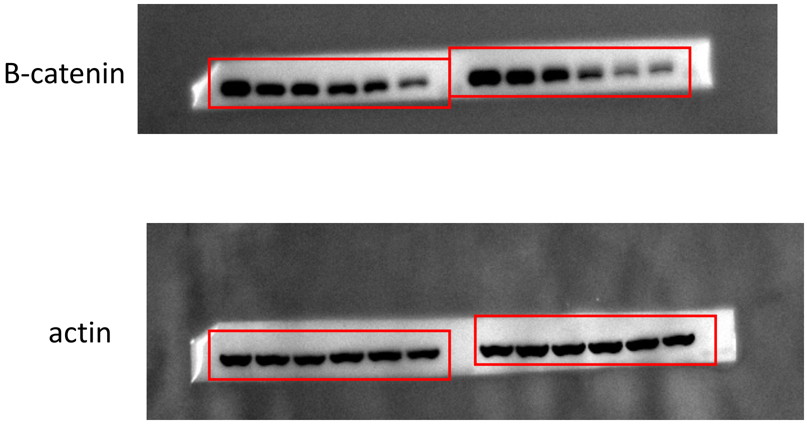

Supplement: Supplementary file 8 — Original Data File [file 41419_2023_5593_MOESM8_ESM.pdf]
